# Synergistic Adsorption and Fluorescence in Porous Aromatic Frameworks for Highly Sensitive Detection of Radioactive Uranium

**DOI:** 10.3390/molecules30091920

**Published:** 2025-04-25

**Authors:** Suming Zhang, Siyu Wu, Cheng Zhang, Doudou Cao, Yingbo Song, Yue Zheng, Jiarui Cao, Lu Luo, Yajie Yang, Xiangjun Zheng, Ye Yuan

**Affiliations:** 1 Beijing Key Laboratory of Energy Conversion and Storage Materials, College of Chemistry, Beijing Normal University, Beijing 100875, China; 2 Key Laboratory of Polyoxometalate and Reticular Material Chemistry of Ministry of Education, Faculty of Chemistry, Northeast Normal University, Changchun 130024, Chinayuany101@nenu.edu.cn (Y.Y.); 3 Key Laboratory of Automobile Materials of Ministry of Education, School of Materials Science and Engineering, Jilin University, Changchun 130022, China

**Keywords:** porous aromatic framework, molecular imprinting, uranyl ion, fluorescence quenching, uranium detection

## Abstract

Uranium plays an important role in the modern nuclear industry. However, such a radioactive element can also cause severe damage to the environment once leaked or discharged into water or air, having a huge impact on the safety of the biosphere. In this work, we pioneered the use of fluorescent monomers as imprinted units, which promoted fluorescence emission of the material. A novel porous aromatic framework was obtained with uranyl ion chelating sites, namely MIPAF-15. The unique N-O chelating pockets on the 4-bromo-1-*H*-indole-7-carboxylic acid gave rise to high coordination affinity toward uranyl ions, which enabled the fast adsorption rate of uranyl ions and a uranyl ion adsorption capacity of 44.88 mg·g^−1^ at 298 K with an initial pH value of 6.0 and the uranyl concentration of 10 ppm. At the same time, the fluorescence quenching effect of MIPAF-15 was observed upon its adsorption of uranyl ions, which allowed the selective detection of uranyl ions with a detection limit of 5.04 × 10^−8^ M, lower than the maximum concentration of uranyl ions in drinking water specified by the World Health Organization (6.30 × 10^−8^ M) and United States Environmental Protection Agency (1.11 × 10^−7^ M). This kind of multifunctional porous material produces a favorable pathway for the detection, removal and degeneration of highly pollutive ions, promoting the overall sustainable development of the natural environment.

## 1. Introduction

Nuclear energy is one of the most attractive energy resources for its high efficiency of energy supply and low emission of greenhouse gases [1,2,3]. Being the most commonly used raw material for the nuclear industry [4], uranium is also a kind of element with extremely high toxicity [5]. Once it enters the human body through water, air or food, uranium will probably deposit and enrich in organs, leading to diseases such as anemia, renal tubular disorders and even cancer [6,7,8,9,10]. And the inappropriate handling of uranium could probably lead to its leakage into the water, as it exists in the form of uranyl ions (UO_2_^2+^) which are highly soluble in water [11,12], becoming a potential hazard to all the living beings on earth. To avoid such destructive effects on human health caused by uranium, a wide range of methods have been developed such as surface adsorption, photoreduction and isotope separation [13,14,15,16], which help the extraction and eradication of uranyl ions. At the same time, the detection of uranyl ions is also of great importance for the protection against and processing of uranium. Instrumental analytical methods are the most proven and widely used methods. Unique physical characteristics such as the radioactivity, atomic absorbance effect and photoelectric effect of uranyl ions helped with the specific detection of it using methods such as electrochemical methods [17,18,19], α-spectroscopy [20], γ-spectroscopy [21], surface-enhanced Raman spectroscopy [22,23,24], liquid scintillation counter (LSC) [25,26], inductively coupled plasma atomic emission spectroscopy (ICP-AES) [27], laser-induced fluorescence analysis (LIF) [28,29], X-ray fluorescence spectroscopy (XRF) [30,31] and so on. Yet, these methods rely on instruments that usually need the pre-treatment of samples, which may bring increased demand for time and labor, hindering their wide application [32]. Thus, the development of uranium detection technology focusing on low time and labor consumption is now a main direction.

In order to address such challenges, a wide range of methods and materials have been developed and utilized in the detection of uranium. Among them, fluorescence sensing has attracted enormous interest because of its fast response, naked-eye visibility, high sensitivity and easy operation [33,34,35,36]. So far, researchers have developed various materials as fluorescence probes for uranyl ions including agars [37,38], ion pairs [39,40], metal complexes [41,42], metal–organic frameworks (MOFs) [43,44,45,46,47,48,49,50], conjugated organic frameworks (COFs) [51,52,53], quantum dots (QDs) [54,55,56], nanoparticles (NPs) [57,58,59], DNAzymes [60] and so on, which showed good capability of uranium detection. Nevertheless, several drawbacks were often observed obstructing the widespread application of the fluorescence probes above. For example, the instability of MOFs and COFs in either a water or acid and base environment remained a great challenge limiting their application scope [61]. In addition, most functional groups used as binding sites of uranyl ions could be affected by many other ions to a great extent owing to the binding of interference ions with coordination sites, such as oxime groups, carboxyl groups, phosphate groups and so on [62]. Thus, there are still significant demands for promoting stability and selectivity in the detection process.

In this study, a novel porous aromatic framework (PAF) constructed by tetrakis (4-ethynylphenyl) methane and 1,3,5-tribromobenzene was synthesized via Sonogashira–Hagihara coupling. In order to provide a suitable coordination configuration for uranyl ions, a uranyl ion complex synthesized by 4-bromo-1-*H*-indole-7-carboxylic acid together with uranyl ion was introduced to the PAF skeleton, obtaining MIPAF-15. Upon the removal of uranyl ions, MIPAF-15 possessed a large specific surface area and good stability and could rapidly adsorb uranyl ions from aqueous solutions. Upon the adsorption of uranyl ions, the fluorescence emission of MIPAF-15 was obviously quenched, which allowed MIPAF-15 to be utilized as a platform for the selective sensing of uranyl ions.

## 2. Results and Discussion

### 2.1. Structural Characterization of MIPAF-15

**Figure 1:**
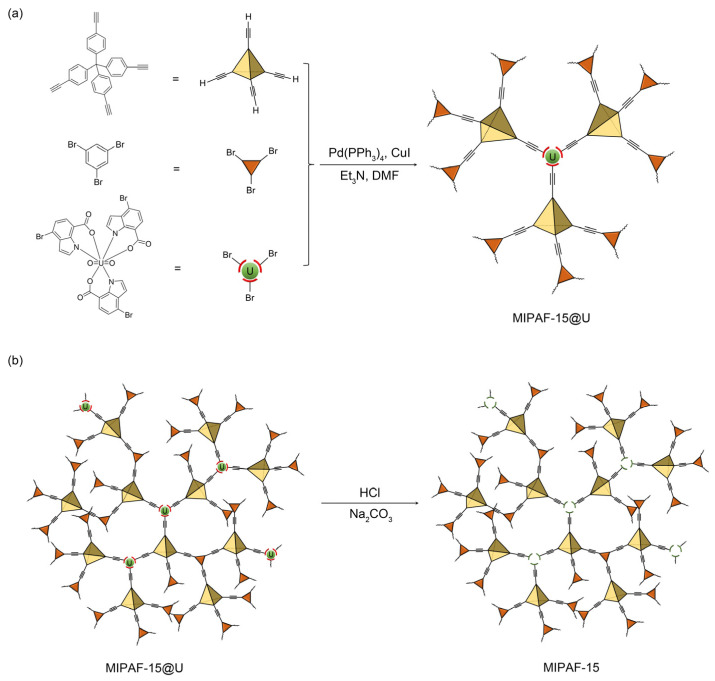
(**a**) Preparation of MIPAF-15@U. (**b**) Release of uranyl ions in MIPAF-15@U.

**Figure 2:**
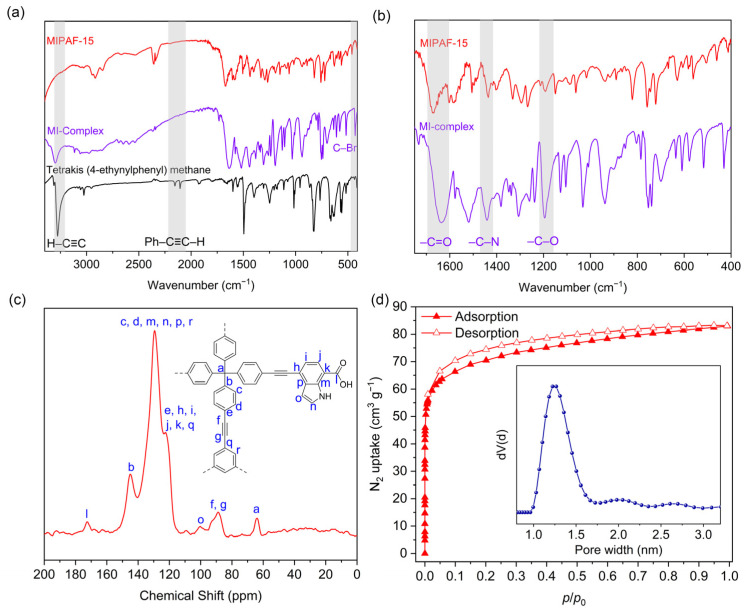
(**a**,**b**) FT-IR spectra of tetrakis (4-ethylphenyl) methane, MI complex, MIPAF-15 and PAF. (**c**) Solid-state ^13^C NMR spectra of MIPAF-15. (**d**) Nitrogen adsorption isotherm and pore-size distribution of MIPAF-15.

Under the catalysis of a palladium (0) and copper (I) catalyst in an alkali environment, C–C bonds were formed between the tetrahedral unit tetrakis (4-ethynylphenyl) methane and the triangular unit 1,3,5-tribromobenzene together with a molecularly imprinted complex (MI-complex) containing uranyl ions, forming a 3D porous framework named MIPAF-15 (Figure 1a). The release of uranyl ions from the MI-complex was realized by treating MIPAF-15 with an aqueous solution of hydrochloric acid and sodium carbonate, exposing coordination sites (Figure 1b). The FT-IR analysis and solid-state ^13^C NMR verified the success of the synthesis. As shown in Figure 2a, the peaks at 410 and 3278 cm^−1^ attributed to C–Br and C≡C–H bonds, respectively, disappeared in MIPAF-15 compared to the MI-Complex, suggesting the successful Sonogashira–Hagihara coupling of building monomers [63,64]. In the zoomed FT-IR spectrum, the emergence of peaks at 1196, 1442 and 1670 cm^−1^ in MIPAF-15 was attributed to the vibrational absorption peaks of C–O, C–N and C=O [63,64], confirming the existence of carboxyl groups and indole rings in MIPAF-15 during the synthesis process (Figure 2b). Solid-state ^13^C NMR of MIPAF-15 showed both the signals of PAF and an indole skeleton. The chemical shifts at 65, 90 and 142 ppm were assigned to alkynyl groups, quaternary carbons and aromatic carbons connected to quaternary carbons, respectively [65,66]. The chemical shifts at 172 and 110 ppm contributed to the carboxyl carbons and sp^2^ carbons of indole [67,68,69,70,71,72] in Figure 2c, which proved the combination of the PAF and MI-Complex. Nitrogen adsorption–desorption tests at 77 K were conducted to verify the specific surface area and pore structure of MIPAF-15. A type-I adsorption isotherm was observed as shown in Figure 2d, indicating that the gas adsorption process of MIPAF-15 followed *Langmuir* monolayer adsorption. The specific surface area of MIPAF-15 was calculated to be 265 m^2^ g^−1^ based on the *Brunauer–Emmett–Teller* (BET) model, which was smaller than some classical PAFs constructed with similar structural units [67,68,69,70,71,72]. Such a reduction in the specific area may be due to the combination of the MI-Complex and PAF skeleton attributing the entrance of the indole fragment to the pore of the PAF [73]. Evaluated by non-localized density functional theory (NL-DFT), MIPAF-15 showed a narrow pore-size distribution at approximately 1.2 nm.

**Figure 3:**
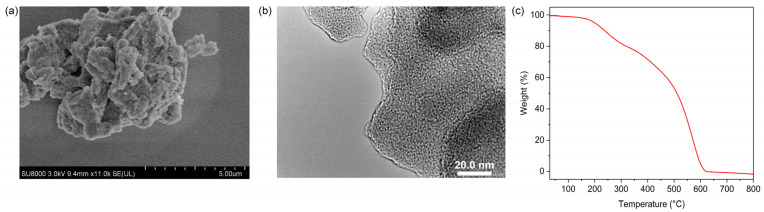
(**a**) SEM image of MIPAF-15. (**b**) TEM image of MIPAF-15. (**c**) Thermogravimetric curve of MIPAF-15.

Scanning (SEM) and transmission electron microscopy (TEM) was used to see the micro-scale structure and morphology of MIPAF-15. As shown in Figure 3a,b, PAF solids aggregated, forming an amorphous structure. As Figure S1 displayed, energy-dispersive X-ray spectroscopy (EDS) mapping images obtained from SEM showed that carbon, oxygen and nitrogen elements uniformly distributed in the MIPAF-15 solid. PXRD pattern gave no significant diffraction peak (Figure S2), showing that MIPAF-15 was in an amorphous phase, like most other PAFs [74,75]. Thermogravimetric analysis showed that there was only a slight weight loss before 200 °C as described in Figure 3c, revealing the high thermal stability of MIPAF-15. Above 300 °C, the polymer began to lose its weight due to the collapse of the organic skeleton.

### 2.2. Uranyl Ion Adsorption Performance of MIPAF-15

**Figure 4:**
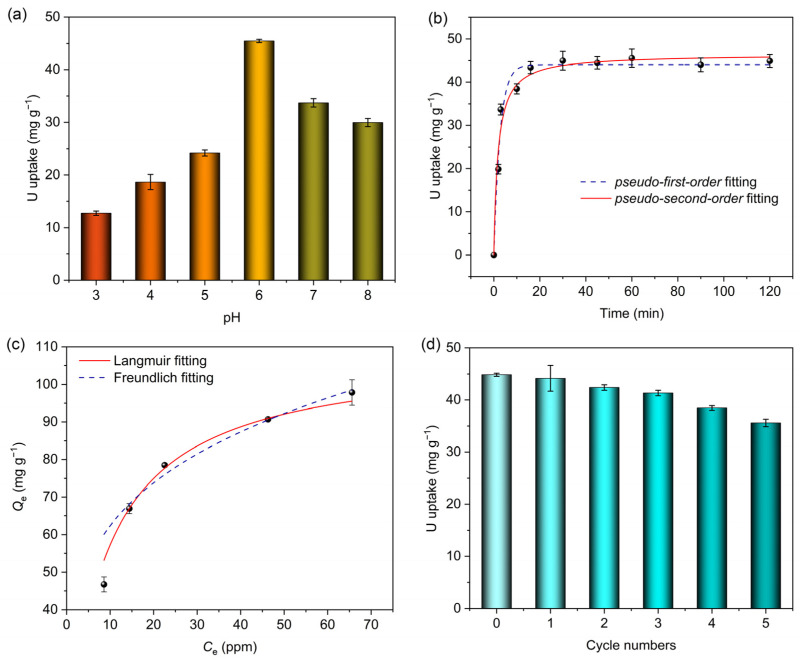
(**a**) Uranium adsorption capacities of MIPAF-15 at different pH values. (**b**) Non-linear fitting of uranium adsorption capacity of MIPAF-15 using *pseudo-first-order* and *pseudo-second-order* models. (**c**) Adsorption isotherm of MIPAF-15 fitted with *Langmuir* and *Freundlich* models. (**d**) Uranium adsorption reusability of MIPAF-15.

As for the adsorption of uranyl ions by porous materials, pH value is a key factor for its significant effect on both the surface properties and the form of existence of uranyl ions. Uranyl ion adsorption experiments with the UO_2_^2+^ concentration of 10 ppm with different pH values ranging from 3 to 8 were carried out. Figure 4a shows that at pH = 6, MIPAF-15 reaches a maximum equilibrium adsorption capacity of 44.88 mg·g^−1^. As the pH value decreased, the equilibrium adsorption capacity decreased sharply, which was possibly caused by the protonation of carboxyl groups connected to the PAF skeleton, resulting in the mask of the coordination sites [73]. Meanwhile, the increase in pH value also resulted in a decrease in equilibrium adsorption capacity. This may be due to the formation of different species of ions of uranium such as (UO_2_)_2_(OH)^2+^, UO_2_(OH)^+^ and so on [48]. Such species with different sizes might be the main reason for the decrease in adsorption capacity when the pH value increases above 6.

At pH = 6, the uranyl adsorption performance of MIPAF-15 in an aqueous solution of uranyl nitrate was tested with a uranyl concentration of 10.00 ppm at room temperature, and the result is displayed in Figure 4b. When the content of MIPAF-15 was 25.00 mg·L^−1^, MIPAF-15 rapidly adsorbed uranyl ions in the first 5 min and reached an equilibrium adsorption capacity of 44.88 mg·g^−1^ within 10 min, showing a high-rate combination of uranyl ions with MIPAF-15. The adsorption kinetics studies were conducted, and the results are shown in Figure 4b. The adsorption capacity at different times was fitted with *pseudo-first-order* and *pseudo-second-order* kinetic models (Figures S3 and S4). The results showed that MIPAF-15 fitted with *pseudo-second-order* kinetic models is better with R^2^ value = 0.9993, revealing that the adsorption process of uranyl ions by MIPAF-15 was mostly dominated by chemical adsorption, that is to say, the coordination of nitrogen atoms and oxygen atoms with uranyl ions. In order to further investigate the adsorption process, *Langmuir* and *Freundlich* models were utilized to fit the adsorption isotherm to reveal the mechanism of adsorption. As Figure 4d shows, the adsorption capacity reached 97.84 mg·g^−1^ as the initial concentration of uranyl ions in the solution increased to 70.0 ppm. The data of fitting showed the linear correlation coefficients were 0.9974 and 0.8917 using *Langmuir* and *Freundlich* models, respectively, as is shown in Figures S5 and S6. The larger value of R^2^ of the fitting with the Langmuir model revealed that monolayer chemisorption played a dominant role in the process of uranyl adsorption by MIPAF-15.

A cyclic stability test of MIPAF-15 was carried out, and the results are shown in Figure 4d. The desorption of uranyl ions from MIPAF-15 was realized by treating uranyl-ion-loaded MIPAF-15 with HCl (0.10 M) and Na_2_CO_3_ (1.00 M) up to three times. After five cycles of the adsorption–desorption experiment, no sharp decrease in adsorption capacity was found, and MIPAF-15 retained 80% of its original adsorption capacity, which indicated the outstanding cyclic stability of MIPAF-15. Meanwhile, MIPAF-15 also showed excellent stability against solvents, sunlight and long-term storage. After it was soaked in HCl, NaOH and ethanol for 4 h, irradiated with sunlight for 12 h or kept in storage for up to 3 months, only a slight reduction in adsorption capacity was observed in MIPAF-15 (Figure S7), revealing its outstanding stability in various conditions.

### 2.3. Fluorescence Sensing Performance of MIPAF-15

**Figure 5:**
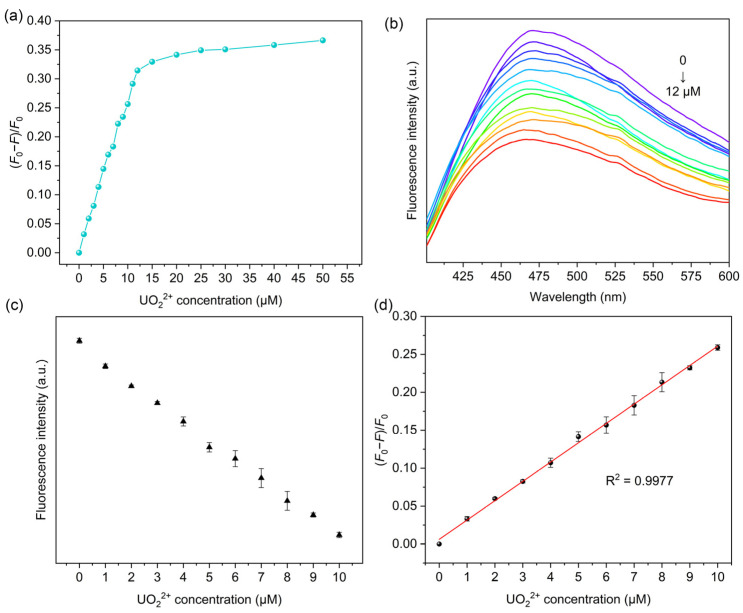
(**a**) Correlation between fluorescence quenching ratio and uranyl ion concentration in deionized water. (**b**) Fluorescence quenching effect of MIPAF-15 at different concentrations of uranyl ion. (**c**) Linear correlation between fluorescence intensity and uranyl ion concentration in deionized water. (**d**) Linear fitting of fluorescence quenching ratio and uranyl ion concentration in deionized water.

In order to examine the fluorescence properties of MIPAF-15 and further study the sensing of uranyl ions, the excitation and emission spectrum of 4-bromo-1-H-indole-7-carboxylic acid (Figure S8) and MIPAF-15 (Figure S9) dispersed in deionized water was measured. Under the excitation of 320 nm, a broad emission of MIPAF-15 with maximum intensity at approximately 470 nm was observed. With the increase in uranyl ions, the fluorescence emission intensity significantly weakened. With 50.00 ppm (approximately 1.85 × 10^−4^ M) of uranyl ions added, about 35% of the initial emission intensity was quenched as shown in Figure 5a,b, and the quenching effect proved to be visible to the naked eye under a 365 nm UV lamp (Figure S10). Given that MIPAF-15 had a strong binding affinity toward uranyl ions, we believe that the coordination of oxygen atoms and nitrogen atoms with uranyl ions was the key to such a dramatic quenching effect on fluorescence emission. In addition, a good linear correlation was observed between the fluorescence quenching ratio [(*F*_0_ − *F*)/*F*_0_], where *F*_0_ represented initial fluorescence intensity measured in deionized water, and *F* represented the fluorescence intensity with different concentrations of uranyl ions added] and concentration of uranyl ions below 10 μM (Figure 5c). The linear correlation coefficient (R^2^) was calculated to be 0.9977, and the linear relationship between the quenching ratio and uranyl ion concentration (μM) could be described as follows:

(*F*_0_ − *F*)/*F*_0_ = 0.02583 *C*(UO_2_^2+^) + 0.00511

**Figure 6:**
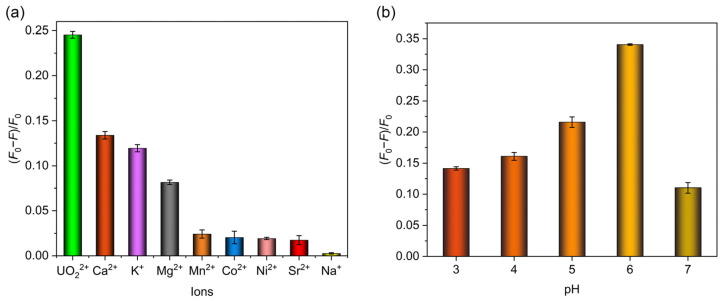
(**a**) Fluorescence quenching selectivity of MIPAF-15. (**b**) Fluorescence quenching rate of MIPAF-15 at different values of pH.

The limit of detection (LOD) was subsequently calculated on the basis of LOD = 3*σ*/*k*, where *σ* represented the standard deviation of blank samples with three parallel measurements (calculated 4.34 × 10^−4^), and *k* represented the slope of a linear equation between the fluorescence quenching ratio and uranyl concentration. The LOD was determined to be 5.04 × 10^−8^ M, which stood below the maximum concentration of uranyl ions in drinking water specified by the World Health Organization (6.30 × 10^−8^ M) and United States Environmental Protection Agency (1.11 × 10^−7^ M) [45,48]. Additionally, the detection performances of some other methods used for uranyl ion detection were compared with MIPAF-15, and the results are listed in Table 1, showing the low LOD of MIPAF-15. Normally, various amounts of other metal ions such as Na^+^, K^+^, Ca^2+^, Mg^2+^ and so on exist in domestic water, river water or seawater. So the influence of other metal ions on the fluorescence quenching and the detection of uranyl ions by MIPAF-15 is always a vital indicator for assessing the detection capability. Due to the high concentration of the ions above, an additional fluorescence quenching ratio was observed with these ions added. To evaluate the influence of interfering ions on the sensing of uranyl ions, fluorescence quenching selectivity tests were carried out. Interfering ions including Na^+^, K^+^, Ca^2+^, Mg^2+^, Sr^2+^, Co^2+^, Mn^2+^ and Ni^2+^ with a concentration of 2.00 × 10^−5^ M were added separately to 5.00 mL deionized water. According to the result shown in Figure 6a, no fluorescence quenching ratio caused by the interfering ions mentioned above could exceed the value caused by 1.00 × 10^−5^ M uranyl ions, indicating the high fluorescence selectivity of MIPAF-15.

As described before, the adsorption capacity of uranyl ions by MIPAF-15 was observed to be greatly affected by pH value. So it was believed that the fluorescence quenching ratio was also determined by pH value. With a uranyl concentration of 10.00 ppm, we measured the fluorescence quenching ratio of MIPAF-15 in pH values ranging from 3 to 7. It was obvious that the quenching ratio peaked at pH = 6 and decreased with higher or lower pH values (Figure 6b). This was consistent with the pH value corresponding to the highest uranyl adsorption capacity. As the pH value got lower, more hydrogen atoms were connected to the carboxyl group, occupying the molecularly imprinted sites and causing a significant decrease in uranium adsorption. Thus, the fluorescence quenching ratio at a pH value below 6 decreased along with the reduction in the adsorption capacity. As the pH value got higher, the main species of uranium in the aqueous solution changed, resulting in a weakened binding affinity of coordination sites toward uranium and a lower fluorescence quenching ratio.

In consideration of the utilization of MIPAF-15 in different practical conditions, fluorescence measurements were conducted using different resources of water, including natural seawater from Bohai and tap water from Nanguan district, Changchun, Jilin Province in China. The same procedure and concentration of the absorbent were used as the former fluorescence quenching experiment was applied, and it was found that in both seawater and tap water, the fluorescence quenching of MIPAF-15 caused by uranyl ions was observed (Figure S11). The linear relationship between the fluorescence quenching ratio and concentration of uranyl ions was fitted, and the limits of detection were calculated to be 6.84 × 10^−8^ M and 9.66 × 10^−8^ M for tap water and seawater, respectively (Figures S12 and S13). Compared to deionized water, the linear range between the fluorescence quenching ratio and concentration of uranyl ions added was greatly shortened, and the detection limits were a little higher in both tap water and seawater. Such a change was caused by the existence of high-concentration ions in real water. As listed in Table 2, the cation with the highest concentration in seawater is Na^+^ with a concentration of approximately 450–470 mM, and the concentrations of Ca^2+^, Mg^2+^ and K^+^ are about 50.00, 10.00 and 9.00 mM, respectively. The high concentration of Ca^2+^, Mg^2+^ and K^+^ could cause the extra quenching effect of MIPAF-15, resulting in an increase in the detection limit toward uranyl ions. Meanwhile, in tap water, the concentrations of Ca^2+^, Mg^2+^, Na^+^ and K^+^ are approximately 2.20, 1.20, 0.80 and 0.50 mM, respectively, all of which are much lower than those in seawater. So, the detection sensitivity in tap water was higher than that in seawater. At the same time, benefiting from the good fluorescence quenching selectivity of MIPAF-15 toward uranyl ions as described before, MIPAF-15 maintained its high sensitivity of detection in real water with complex ion consistency and effectively reduced the impact of matrix effects, promoting its high potential of practical application (Figure S14). Thus, MIPAF-15 was capable of detecting uranyl ions in a wide range of circumstances based on the selective fluorescence quenching effect caused by uranyl ions.

To further investigate the coordination mode of uranyl ions on MIPAF-15 and clarify the quenching mechanism of uranyl ions on the fluorescence of MIPAF-15, X-ray photoelectron spectroscopy was carried out. After the adsorption, new peaks at 383.1 and 393.9 eV emerged, belonging to U_4f 7/2_ and U_4f 5/2_, respectively, and were lower than those in uncoordinated uranyl ions, proving the binding of uranyl ions (Figures S15 and S16). The O_1s_ peak significantly shifted toward higher binding energy from 531.8 to 532.2 eV after the adsorption of uranyl ions, indicating the chelating of uranyl ions with oxygen atoms from carboxyl groups [77]. In the high-resolution XPS spectra of MIPAF-15 (Figure S17), the O_1s_ peak could be divided into two peaks at 531.4 and 532.9 eV. The former was attributed to COO^−^, and the latter corresponded to H–O. After the adsorption of uranyl ions, the peak of COO^−^ shifted to a higher binding energy of 532.1 eV, and a new peak at approximately 533.4 eV attributed to O=U=O appeared [78,79]. Moreover, in the high-resolution N_1s_ XPS spectra, after the adsorption of uranyl ions, the peak at 400.1 eV belonging to C–N shifted to 400.2 eV, and a new peak belonging to N–U located at 401.0 eV appeared in Figure S18 [51], indicating the participation of nitrogen atoms in the coordination with uranyl ions. These alternations in the XPS peaks confirmed that both the oxygen atoms from carboxyl groups and the nitrogen atoms from indole rings participated in the coordination with uranyl ions. Considering the high affinity and selectivity to uranyl ions, it could obviously be attributed to the evolvement of coordination sites with a suitable size for uranyl ions, which caused the good selectivity of coordination between MIPAF-15 and uranyl ions. In addition, the stronger energy transfer between uranyl ions and MIPAF-15 caused by the special electron and spatial configuration of uranyl ions may also contribute to the outstanding fluorescence quenching effect of uranyl ions on MIPAF-15 [76].

## 3. Materials and Methods

### 3.1. Materials

All starting materials are commercially available and used without further purification.

Tetrakis (4-ethynylphenyl) methane (Leyan, Shanghai, China, 97%), 1,3,5-tribromobenzene (TCI, Tokyo, Japan, 98%), uranyl nitrate hexahydrate (Aladdin, Shanghai, China, AR), 4-bromo-1-*H*-indole-7-carboxylic acid (Leyan, 97%), copper(I) iodide (Leyan, 99%), tetrakis(triphenylphosphine) palladium (0) (Aladdin, 99%), sodium carbonate anhydrous (Aladdin, AR), sodium chloride (Aladdin, AR), calcium chloride anhydrous (Aladdin, AR), potassium nitrate (Aladdin, AR), magnesium chloride hexahydrate (Aladdin, AR), manganese (II) chloride anhydrous (Aladdin, AR), cobalt (II) chloride hexahydrate (Aladdin, AR), nickel chloride hexahydrate (Aladdin, AR), strontium chloride hexahydrate (Aladdin, AR), *N*,*N*-dimethylformamide (Damas-beta, Shanghai, China, anhydrous, with molecular sieve), triethylamine (Aladdin, anhydrous, with molecular sieve), methanol (Shanghai Chemical Reagents, Shanghai, China, AR), ethanol (Shanghai Chemical Reagents, AR), tetrahydrofuran (Shanghai Chemical Reagents, AR), trichloromethane (Shanghai Chemical Reagents, AR), hydrochloric acid (Shanghai Chemical Reagents, 37% aqueous solution), acetone (Shanghai Chemical Reagents, AR).

### 3.2. Instrumentation and Physical Measurements

Fourier transform infrared spectroscopy (FT-IR) was performed on Nicolet Impact 410 (Madison, WI, USA). Solid-state ^13^C nuclear magnetic resonance (Solid-state ^13^C NMR) data were recorded using Bruker Avance NEO 400 (Billerica, MA, USA). Powder X-ray diffraction (PXRD) was obtained on a Bruker D8 QUEST diffractometer (Saarbrücken, German) with Cu-Kα radiation. Nitrogen adsorption–desorption measurement was performed on a Quantachrome Auto-sorb (Boynton Beach, FL, USA). Scanning electron microscopy (SEM) images and energy-dispersive X-ray spectroscopy (EDS) mapping images were obtained with JEOL JSM 6700 (Akishima, Japan), and transmission electron microscope (TEM) images were obtained on JEM-2100 (Akishima, Japan). Thermogravimetric analysis (TGA) was measured by a Mettler-Toledo TGA/DSC 2 thermal analyzer (Zurich, Switzerland) under an air atmosphere at a heating rate of 10 °C min^−1^. X-ray photoelectron spectroscopy (XPS) was measured using AXIS ULTRA (Lund, Sweden). UV-vis absorption spectra. UV-vis absorbance spectroscopy was conducted with VARIAN Cary500 (Palo Alto, CA, USA). Fluorescence properties were measured using a Shimadzu RF-6000 fluorescence spectrophotometer (Kyoto, Japan).

### 3.3. Synthesis

#### 3.3.1. Synthesis of Molecularly Imprinted Complex

To synthesize a molecularly imprinted complex (MI-complex), 288.0 mg of 4-bromo-1-*H*-indole-7-carboxylic acid (1.20 mmol) was dissolved in 15.00 mL ethanol and stirred. Then, 200.0 mg of uranyl nitrate hexahydrate (0.40 mmol) was added to the solution, further stirred at room temperature for 4 h and then allowed to reflux at 80 °C for an additional 2 h, obtaining a light yellow solution. The solvent was further removed by rotary evaporation, and the obtained light-brown solid was allowed to be washed with deionized water and dried in vacuum under 50 °C for 12 h. Yield 351.5 mg (89.07%).

#### 3.3.2. Synthesis of Uranyl Ion Imprinted Porous Aromatic Framework

To synthesize a uranyl ion imprinted porous aromatic framework named MIPAF-15, Sonogashira–Hagihara coupling was applied, and the procedure was optimized based on previous literature [63,65]. A total of 125.0 mg of tetrakis (4-ethynylphenyl) methane (0.30 mmol), 113.4 mg of 1,3,5-tribromobenzene (0.32 mmol) and 79.0 mg of MI-complex (0.08 mmol) was added into a two-necked flask. Next, 10.00 mL of anhydrous N, N-dimethylformamide and 10.00 mL of anhydrous triethylamine were added. Then, 10.0 mg of copper(I) iodide and 25.0 mg of tetrakis(triphenylphosphine) palladium (0) were added as catalysts of the coupling reaction. Followed by 3 times of the sequence of freezing–vacuuming–thawing, the flask was filled with nitrogen. Then, the mixture was heated to 120 °C in an oil bath and stirred for 72 h. Upon heating, a light yellow solid precipitated from the reaction mixture, and the color turned to dark brown. The obtained dark-brown solid was then sequentially washed with methanol, HCl (1.00 M, aq), acetone, trichloromethane, N, N-dimethylformamide and tetrahydrofuran for 1 h each, followed by Soxhlet extraction with methanol for 72 h. To remove the uranyl ions in the imprinted coordination sites, the obtained solid was further stirred in HCl (0.10 M, aq) and Na_2_CO_3_ (1.00 M, aq) as many times as needed in order to unload uranyl ions from the imprinted coordination sites and obtain uranyl ion imprinted porous aromatic framework. Yield 178.9 mg (96.60%).

### 3.4. Uranyl Ion Adsorption Experiment

The solid of MIPAF-15 was dispersed in a 10.00 ppm aqueous solution of uranyl nitrate, and the content of the absorbent was 25.00 mg L^−1^. The original pH value of uranyl nitrate was adjusted by HCl (0.10 M) and Na_2_CO_3_ (1.00 M), measured to be 6. Then, the mixture was constantly stirred while the supernatant of different times was collected for the following measurement of uranyl concentration using the formula 1.

(1) $$Q_{e}=\frac{V\left(C_{0}-C_{e}\right)}{m}$$

where *Q*_e_ represents the uranium adsorption capacity of the sorbent (mg g^−1^), *V* represents the volume of the uranyl nitrate solution (L), *C*_0_ represents the initial concentration of uranyl ions (mg L^−1^), *C*_e_ represents the equilibrium concentration of uranyl ions (mg L^−1^), and *m* represents the mass of the sorbent (g).

Uranium adsorption kinetics were fitted by using *pseudo-first-order* and *pseudo-second-order* kinetic models as Formulas (2) and (3).

(2) $$ln{\left(Q_{e}-Q_{t}\right)=lnQ}_{e}-k_{1}t$$

(3) $$\frac{t}{Q_{t}}=\frac{1}{k_{2}Q_{e}^{2}}+\frac{t}{Q_{e}}$$

where *Q*_e_ represents the equilibrium adsorption capacity (mg g^−1^), *Q*_t_ represents the adsorption capacity at a certain time (mg g^−1^), *k*_1_ and *k*_2_ represent first-order and second-order adsorption constant, respectively, and *t* represents time (s).

Fittings of the adsorption isotherm were carried out using the Langmuir model and Freundlich model as Formulas (4) and (5).

(4) $$\frac{C_{e}}{Q_{e}}=\frac{C_{e}}{Q_{max}}+\frac{1}{k_{L}Q_{max}}$$

(5) $$lnQ_{e}=lnk_{F}+\frac{lnC_{e}}{n}$$

where *Q*_e_ represents the equilibrium adsorption capacity (mg g^−1^), *C*_e_ represents the equilibrium concentration (mg L^−1^), *Q*_max_ represents the maximum adsorption capacity (mg g^−1^), *k*_L_ and *k*_F_ represent the Langmuir and Freundlich constants, and n represents adsorption capacity.

### 3.5. Fluorescence Detection of Uranyl Ion

Considering the strong chelating ability of carboxyl acid groups together with nitrogen atoms and the rapid adsorption of uranyl ions, MIPAF-15 was employed as a type of fluorescence probe for uranyl ions. A total of 1.0 mg of MIPAF-15 was dispersed in 5.00 mL of aqueous solution with pH value = 6 and different concentrations of UO_2_^2+^, followed by ultrasonication for up to 30 s. With the formation of a uniform suspension, the fluorescence spectrum was measured.

## 4. Conclusions

In summary, we successfully synthesized a novel porous aromatic framework with the first combination of molecular-imprinted coordination sites with ligands of fluorescence emission. The resulting MIPAF-15 polymer was constructed by Sonogashira–Hagihara coupling reaction using tetrahedral and triangular structural units and a molecularly imprinted complex composed of uranyl ions and 4-bromo-1-*H*-indole-7-carboxylic acid. Attributed to the involvement of carboxyl groups and indole rings into the PAF skeleton, MIPAF-15 showed good affinity toward uranyl ions and rapid uptake of uranyl ions from water. After the adsorption of uranyl ions, the fluorescence properties of MIPAF-15 changed greatly caused by energy transfer between uranyl ions and the PAF skeleton. MIPAF-15 was also able to be used as a uranyl detector in real water such as seawater and tap water, which provided a wide avenue for the removal and recognition of pollutants in our environment.

## Figures and Tables

**Table 1:** Comparisons of detection performances of some classical methods applied for the detection of uranyl ions.

Reagent	pH	Usage (per L Water)	Contact Time	LOD (nM)	Cost	Reference
Eu-MOF	7.0	0.20 g	immediately	900.00	USD 30.56/g	[46]
Au-nanoparticles	7.0	0.50 L	several minutes	84.00	USD 406.38/L	[58]
Zn-MOF	3.0	0.50 g	immediately	12.00	USD 15.32/g	[48]
Eu-complex	7.4	0.54 g	immediately	12,000	USD 22.35/g	[41]
N-doped QD	7.0	0.01 g	3 h	20.38	USD 241.70/g	[56]
AO-CMP	6.0	0.20 g	3 h	1.70	USD 831.89/g	[76]
MIPAF-15	6.0	0.20 g	30 s	50.40	USD 150.51/g	This work

**Table 2:** Concentration of cations in selected real water.

Cations	Tap Water (mM)	Seawater (mM)
Na^+^	0.80	450.00–470.00
Ca^2+^	2.20	50.00
K^+^	0.50	10.00
Mg^2+^	1.20	9.00

## Data Availability

All data related to the study are presented in the main text and Supplementary Materials.

## Supplementary Materials

The following supporting information can be downloaded at https://www.mdpi.com/article/10.3390/molecules30091920/s1, Figure S1: EDS images of MIPAF-15; Figure S2: PXRD pattern of MIPAF-15; Figure S3: Kinetic fitting with *pseudo-first-order* model; Figure S4: Kinetic fitting with *pseudo-second-order* model; Figure S5: Fitting of adsorption isotherm with *Langmuir* model; Figure S6: Fitting of adsorption isotherm with *Freundlich* model; Figure S7: Stability tests of MIPAF-15 against different conditions; Figure S8: Fluorescence excitation and emission spectra of 4-bromo-1-*H*-indole-7-carboxylic acid; Figure S9: Fluorescence excitation and emission spectra of MIPAF-15; Figure S10: Photograph of MIPAF-15 with absence (left) and presence (right) of uranyl ions under 365 nm UV lamp; Figure S11: Correlation between fluorescence quenching ratio and uranyl ion concentration in selected natural water; Figure S12: Linear fitting between fluorescence quenching ratio and uranyl ion concentration in tap water; Figure S13: Linear fitting between fluorescence quenching ratio and uranyl ion concentration in seawater; Figure S14: Detection limits of MIPAF-15 towards uranyl ions in different real water; Figure S15: XPS spectra of MIPAF-15 before and after the adsorption of uranyl ions; Figure S16: U_4f_ XPS spectra of uranyl-loaded MIPAF-15; Figure S17: O_1s_ XPS spectra of MIPAF-15 before and after the adsorption of uranyl ions; Figure S18: N_1s_ XPS spectra of MIPAF-15 before and after the adsorption of uranyl ions.

## References

[1] Chu S., Majumdar A. (2012). Opportunities and challenges for a sustainable energy future. Nature.

[2] Sholl D.S., Lively R.P. (2016). Seven chemical separations to change the world. Nature.

[3] Yuan Y., Cao D.D., Cui F.C., Yang Y.J., Zhang C., Song Y.B., Zheng Y., Cao J.R., Chen S.S., Song Y. (2025). High-capacity uranium extraction from seawater through constructing synergistic multiple dynamic bonds. Nat. Water.

[4] Yin X.J., Bai J., Fan F.L., Cheng W.W., Tian W., Wang Y., Qin Z. (2015). Amidoximed silica for uranium (VI) sorption from aqueous solution. J. Radioanal. Nucl. Chem..

[5] Bleise A., Danesi P.R., Burkart W. (2003). Properties, use and health effects of depleted uranium (DU): A general overview. J. Environ. Radioact..

[6] Agius R., Batistatou E., Gittins M., Jones S., McNamee R., Liu H., Rashid A., van Tongeren M., von Oertzen G., Wakeford R. (2023). An epidemiological study of lung cancer and selected other cancers among Namibian uranium workers. Radiat. Res..

[7] Ahmed R.S., Mohammed R.S., Mahdi K.H., Mahdi Q.A., Mostafa M.Y., Khalaf H.N. (2022). Evaluation of uranium concentration in the blood breast cancer women with CR-39 detector. Appl. Radiat. Isot..

[8] Abdulrudha N.H., Kadhim S.A. (2025). The relationship of cadmium, lead, and uranium with the geographical location of non-smoking thalassemia individuals: A comparative study. Appl. Radiat. Isot..

[9] Berradi H., Bertho J., Dudoignon N., Mazur A., Grandcolas L., Baudelin C., Grison S., Voisin P., Gourmelon P., Dublineau I. (2008). Renal anemia induced by chronic ingestion of depleted uranium in rats. Toxicol. Sci..

[10] Homma-Takeda S., Fujishiro H., Tanaka I., Yakumaru H., Ayama K., Uehara A., Oikawa M., Himeno S., Ishihara H. (2021). Single-cell imaging for studies of renal uranium transport and intracellular behavior. Minerals.

[11] Xiao F.B., Sun Y.F., Du W.F., Shi W.H., Wu Y., Liao S.Z., Wu Z.Y., Yu R.Q. (2017). Smart photonic crystal hydrogel material for uranyl ion monitoring and removal in water. Adv. Funct. Mater..

[12] Cooper K.L., Dashner E.J., Tsosie R., Cho Y.M., Lewis J., Hudson L.G. (2016). Inhibition of poly (ADP-ribose) polymerase-1 and DNA repair by uranium. Toxicol. Appl. Pharmacol..

[13] Zhu J.H., Wang J., Liu Q., Yu J., Liu J.Y., Chen R.R., Song D.L., Li R.M., Wang J. (2025). Amidoxime-functionalized MXene/graphene oxide aerogel for sunlight enhanced uranium adsorption. J. Environ. Chem. Eng..

[14] Burns H.S., Biegalski S.R. (2022). Forensic signatures from laser isotope separation. J. Radioanal. Nucl. Chem..

[15] Chen D.Y., Sun M.F., Zhao X.Y., Shi M.S., Fu X.Y., Hu W., Zhao R. (2024). High-efficiency and economical uranium extraction from seawater with easily prepared supramolecular complexes. J. Colloid Interface. Sci..

[16] Yang Y.L., Guo K.K., Zhu M.C., Zhang A.G., Xing M., Lu Y., Bai X., Ji X.Y., Hu Y.J., Liu S.X. (2024). Exploring electron transfer mechanism in synergistic interactional reduced polyoxometalate-based Cu(I)–organic framework for photocatalytic removal of U(VI). Inorg. Chem..

[17] Ali T.A., Akl Z.F. (2021). Ionic liquid-multi-walled carbon nanotubes modified screen-printed electrodes for sensitive electrochemical sensing of uranium. J. Radioanal. Nucl. Chem..

[18] Korolkov I.V., Yeszhanov A.B., Shakayeva A.K., Shlimas D.I., Zhumazhanova A., Zdorovets M.V. (2022). Photo-induced graft (co)polymerization of glycidyl methacrylate and acrylonitrile on PET ion-track membranes for electrochemical detection of uranyl ions. Colloids Surf. A.

[19] Kersten B., Akolkar R., Duval C.E. (2023). An electrochemical technique for sensing uranium adsorption and desorption. Anal. Chim. Acta.

[20] Khaing H., Lemons B.G., Thakur P. (2016). Concentration of uranium in the drinking and surface water around the WIPP site. J. Radioanal. Nucl. Chem..

[21] Dodd B., Cartwright M., Goddard B., Tepper G. (2018). Investigation of uranium (VI) sorption in mesoporous silica gel using gamma spectroscopy. J. Radioanal. Nucl. Chem..

[22] Jiang J.L., Du Y.F., Deng H., Zhang Z.J., Wang S.F., Wu H.X., Tang H., Yun W., Zhang J., He W.B. (2022). Surface-enhanced Raman spectroscopy detection of uranium oxides assisted by Ag_2_O. Appl. Surf. Sci..

[23] Pointurier F., Marie O. (2023). An optimized methodology for the determination of the uranium chemical phases in micro-particles by Raman spectrometry within a scanning electron microscope. J. Radioanal. Nucl. Chem..

[24] Wang N., Du J.J., Li X., Ji X.L., Wu Y.L., Sun Z.L. (2023). Magnetic MOF substrates for the rapid and sensitive surface-enhanced Raman scattering detection of uranyl. Anal. Chem..

[25] Cao X.Y., Lv N., Lv J.X., Guo H.P. (2021). A liquid scintillation analysis method for low-level radioactive wastewater. J. Radiol. Prot..

[26] Pulhani V., Reddy P.J., Chaudhury M., Tripathi R.M. (2019). Sequential analysis methodology for ^210^Po and uranium analysis by extractive liquid scintillation spectrometry. J. Radioanal. Nucl. Chem..

[27] Déjeant A., Bourva L., Sia R., Galoisy L., Calas G., Phrommavanh V., Descostes M. (2014). Field analyses of ^238^U and ^226^Ra in two uranium mill tailings piles from Niger using portable HPGe detector. J. Environ. Radioact..

[28] Zavadilová A., Drtinová B. (2015). The matrix influence on the determination of low uranium concentrations by laser induced fluorescence method. J. Radioanal. Nucl. Chem..

[29] Oba M., Akaoka K., Miyabe M., Wakaida I. (2000). Zeeman effect of atomic uranium in the high lying odd levels measured by laser induced fluorescence spectroscopy. Eur. Phys. J. D.

[30] Fang Y.T., Zhang Z.Q., Hou L.D., Wang Z.I., Zhao Y.P., Liu Q.W., Liu S.W., Chang X.L., Qin Y.Q., Ma J. (2025). Determination of trace uranium concentrations in spent-fuel reprocessing using online graphite crystal pre-diffraction energy-dispersive X-ray fluorescence. X-Ray Spectrom..

[31] Takahashi H., Izumoto Y., Matsuyama T., Yoshii H. (2019). Trace determination of uranium preconcentrated using graphene oxide by total reflection X-ray fluorescence spectrometry. X-Ray Spectrom..

[32] Devore M.A., Kerns S.A., Gorden A.E.V. (2015). Characterization of quinoxolinol salen ligands as selective ligands for chemosensors for uranium. Eur. J. Inorg. Chem..

[33] Feng T.T., Zhao S.L., Cao M., Du X.F., Wang H., Cao X.W., Feng L.J., Yuan Y.H., Wang N. (2025). Highly sensitive and specific uranyl ion detection by a fluorescent sensor containing uranyl-specific recognition sites. Sci. Bull..

[34] Xiong L.H., Tong Y.Q., Song J.Y., Chen S.H., Liu Y., Liu J.Q., Li L., Zhen D.S. (2025). Smartphone-assisted fluorescence/colorimetric dual-mode sensing strategy for uranium ion detection using cerium-sulfonyl calix [4] arene. Microchim. Acta.

[35] Xia Z., Shi Y., Xiao T., Zheng X. (2024). Multi-stimuli responsive behavior of two Zn (II) complexes based on a Schiff base with high contrast. CrystEngComm.

[36] Xiao T., Yang D.D., Shi Y.S., Zheng H.W., Xia Z.G., Zheng X.J. (2023). Hydrazone-based europium (III) complexes: Mechanochromic luminescence and turn-on fluorescence detection of quinolone antibiotics in human urine. Cryst. Growth Des..

[37] Liu H.J., Wang X.L., Abeywickrama T., Jahanbazi F., Min Z.F., Lee Z.R., Terry J., Mao Y.B. (2021). Biomimetically synthesized luminescent Tb^3+^ -doped fluorapatite/agar nanocomposite for detecting UO_2_^2+^, Cu^2+^, and Cr^3+^ ions. Environ. Sci. Nano.

[38] Liu H.J., Wang X.L., Xiong W.J., Mao Y.B. (2022). Luminescent Tb-doped Ca-deficient hydroxyapatite/agar for selective adsorption and detection of UO_2_^2+^ ion. Mater. Res. Bull..

[39] Su Z., Zhang L.X., Zhang H.Q., Li Y.S., Guan Q.Q. (2025). Biplane ion-pairing induced supramolecular assembly for high-performance uranium detection. Adv. Mater..

[40] Zhou X.Y., Wang Y., Xiong L.H., Song J.Y., Zhou H., Li L., Zhen D.S. (2025). Development of rare earth europium composites for highly sensitive fluorescence enhancement for detection of uranyl ions in water and cells. J. Radioanal. Nucl. Chem..

[41] Harvey P., Nonat A., Platas Iglesias C., Natrajan L.S., Charbonnière L.J. (2018). Sensing uranyl (VI) ions by coordination and energy transfer to a luminescent europium (III) complex. Angew. Chem. Int. Ed..

[42] Jiang M., Xiao X., He B., Liu Y., Hu N., Su C.L., Li Z.Y., Liao L.F. (2019). A europium (III) complex-based surface fluorescence sensor for the determination of uranium (VI). J. Radioanal. Nucl. Chem..

[43] Chen W.M., Meng X.L., Zhuang G.L., Wang Z., Kurmoo M., Zhao Q.Q., Wang X.P., Shan B., Tung C.H., Sun D. (2017). A superior fluorescent sensor for Al^3+^ and UO_2_
^2+^ based on a Co (II) metal–organic framework with exposed pyrimidyl Lewis base sites. J. Mater. Chem. A.

[44] Rapti S., Sarma D., Diamantis S.A., Skliri E., Armatas G.S., Tsipis A.C., Hassan Y.S., Alkordi M., Malliakas C.D., Kanatzidis M.G. (2017). All in one porous material: Exceptional sorption and selective sensing of hexavalent chromium by using a Zr^4+^ MOF. J. Mater. Chem. A.

[45] Liu W., Dai X., Bai Z.L., Wang Y.L., Yang Z.X., Zhang L.J., Xu L., Chen L.H., Li Y.X., Gui D.X. (2017). Highly sensitive and selective uranium detection in natural water systems using a luminescent mesoporous metal–organic framework equipped with abundant Lewis basic sites: A combined batch, X-ray absorption spectroscopy, and first principles simulation investigation. Environ. Sci. Technol..

[46] Li L.N., Shen S.S., Su J., Ai W.P., Bai Y., Liu H.W. (2019). Facile one-step solvothermal synthesis of a luminescent europium metal-organic framework for rapid and selective sensing of uranyl ions. Anal. Bioanal. Chem..

[47] Chen N.N., Wang J. (2020). A serial of 2D Co-Zn isomorphous metal–organic frameworks for photodegradation and luminescent detection properties. Appl. Organomet. Chem..

[48] Qin X.D., Yang W.T., Yang Y.H., Gu D.X., Guo D.Y., Pan Q.H. (2020). A zinc metal–organic framework for concurrent adsorption and detection of uranium. Inorg. Chem..

[49] Feng H., Feng X.F., Luo F. (2021). A 1D brick-like coordination polymer containing free-standing sulfonic units for luminescence sensing of uranium in aqueous solution. J. Solid State Chem..

[50] Huang Z.W., Li X.B., Mei L., Han Y.Z., Song Y.T., Fu X., Zhang Z.H., Guo Z.J., Zeng J.R., Bian F.G. (2024). All-in-one: Photo-responsive lanthanide-organic framework for simultaneous sensing, adsorption, and photocatalytic reduction of uranium. Adv. Funct. Mater..

[51] Cui W.R., Zhang C.R., Jiang W., Li F.F., Liang R.P., Liu J.W., Qiu J.D. (2020). Regenerable and stable sp^2^ carbon-conjugated covalent organic frameworks for selective detection and extraction of uranium. Nat. Commun..

[52] Xiao S.J., Qiu A.T., Li H.H., Wang M.P., Zhang L., Guo K.X., Guo J., Qiu J. (2023). Simultaneous detection and separation of uranium based on a fluorescent amidoxime-functionalized covalent organic polymer. Spectrochim. Acta Part A.

[53] Mao X.L., Cai Y.J., Luo Q.X., Liu X., Jiang Q.Q., Zhang C.R., Zhang L., Liang R.P., Qiu J.D. (2024). Europium (III) functionalized covalent organic framework as sensitive and selective fluorescent switch for detection of uranium. Anal. Chem..

[54] Bhanjana G., Toor I., Chaudhary G.R., Dilbaghi N., Kim K., Kumar S. (2019). Direct redox sensing of uranium using copper oxide quantum dots. J. Mol. Liq..

[55] Singhal P., Pulhani V. (2019). Effect of ligand concentration, dilution, and excitation wavelength on the emission properties of CdSe/CdS core shell quantum dots and their implication on detection of uranium. Chem. Slct..

[56] Wang Q., Zhang H.Y., Yu D.M., Qin W., Wu X.H. (2022). Ultra-sensitive and stable n-doped carbon dots for selective detection of uranium through electron transfer induced UO_2_^+^ (V) sensing mechanism. Carbon.

[57] Yang M., Liao L.F., Zhang G.L., He B., Xiao X.L., Lin Y.W., Nie C.M. (2013). Detection of uranium with a wireless sensing method by using salophen as receptor and magnetic nanoparticles as signal-amplifying tags. J. Radioanal. Nucl. Chem..

[58] Cao X.H., Zhang H.Y., Ma R.C., Yang Q., Zhang Z.B., Liu Y.H. (2015). Visual colorimetric detection of UO_2_^2+^ using o-phosphorylethanolamine-functionalized gold nanoparticles. Sens. Actuators B.

[59] Li W.L., Mayo J.T., Benoit D.N., Troyer L.D., Lewicka Z.A., Lafferty B.J., Catalano J.G., Lee S.S., Colvin V.L., Fortner J.D. (2016). Engineered superparamagnetic iron oxide nanoparticles for ultra-enhanced uranium separation and sensing. J. Mater. Chem. A.

[60] Wang X.L., Zeng R., Chu S.N., Tang W., Lin N., Fu J., Yang J.R., Gao B. (2019). A quencher-free DNAzyme beacon for fluorescently sensing uranyl ions via embedding 2-aminopurine. Biosens. Bioelectron..

[61] Yuan Y., Zhu G.S. (2019). Porous aromatic frameworks as a platform for multifunctional applications. ACS Cent. Sci..

[62] Yuan Y., Meng Q.H., Faheem M., Yang Y.J., Li Z., Wang Z.Y., Deng D., Sun F.X., He H.M., Huang Y.H. (2019). A molecular coordination template strategy for designing selective porous aromatic framework materials for uranyl capture. ACS Cent. Sci..

[63] Zhou S., Liu Z.L., Zhang P.P., Rong H.Z., Ma T.T., Cui F.C., Liu D.T., Zou X.Q., Zhu G.S. (2022). Tailoring the pore chemistry in porous aromatic frameworks for selective separation of acetylene from ethylene. Chem. Sci..

[64] Jang J.Y., Kim Y.K., Ko Y., Son S.U. (2024). Hollow microporous organic polymer@hypercrosslinked polymer catalysts bearing *N* -heterocyclic carbene–iron species for the synthesis of biomass-derived polymer platforms. ACS Sustain. Chem. Eng..

[65] Ma H.P., Ren H., Zou X.Q., Sun F.X., Yan Z.J., Cai K., Wang D.Y., Zhu G.S. (2013). Novel lithium-loaded porous aromatic framework for efficient CO_2_ and H_2_ uptake. J. Mater. Chem. A.

[66] Li X., Cui Y., Yang C., Yan X. (2020). Synthesis of carboxyl functionalized microporous organic network for solid phase extraction coupled with high-performance liquid chromatography for the determination of phenols in water samples. Talanta.

[67] Kumar K., Zapf A., Michalik D., Tillack A., Heinrich T., Böttcher H., Arlt M., Beller M. (2004). Palladium-catalyzed carbonylation of haloindoles:  No need for protecting groups. Org. Lett..

[68] Mistry S.N., Shonberg J., Draper-Joyce C.J., Klein Herenbrink C., Michino M., Shi L., Christopoulos A., Capuano B., Scammells P.J., Lane J.R. (2015). Discovery of a novel class of negative allosteric modulator of the dopamine d_2_ receptor through fragmentation of a bitopic ligand. J. Med. Chem..

[69] Park S.I., Kang C.W., Cho S.Y., Lee S.M., Kim H.J., Ko Y., Choi J., Son S.U. (2020). Fabrication of poly (ethylene terephthalate) fiber @ microporous organic polymer with amino groups @ Cu films for flexible and metal-economical electromagnetic interference shielding materials. Langmuir.

[70] Sk M., Saifi S., Bera S., Ghosh A., Aijaz A., Banerjee D. (2025). Reusable Ni-immobilized MOF catalyst for dehydrogenation of n-heterocycles under milder conditions. Chem. Eur. J..

[71] Yan Z.J., Cui B., Zhao T., Luo Y.F., Zhang H.C., Xie J.L., Li N., Bu N.S., Yuan Y., Xia L.X. (2021). A carbazole-functionalized porous aromatic framework for enhancing volatile iodine capture via Lewis electron pairing. Molecules.

[72] Yan Z.J., Qiao Y.M., Wang J.L., Xie J.L., Cui B., Fu Y., Lu J.W., Yang Y.J., Bu N.S., Yuan Y. (2022). An azo-group-functionalized porous aromatic framework for achieving highly efficient capture of iodine. Molecules.

[73] Yang Y.J., Cai F.L., Zhang C., Gao N., Zhang S.M., Wang G.T., Yuan Y. (2024). Molecularly imprinted porous-organic framework with pH-responsive adsorption sites for the selective adsorption of iron. Chin. J. Chem..

[74] Ben T., Ren H., Ma S.Q., Cao D.P., Lan J.H., Jing X.F., Wang W.C., Xu J., Deng F., Simmons J.M. (2009). Targeted synthesis of a porous aromatic framework with high stability and exceptionally high surface area. Angew. Chem. Int. Ed..

[75] Yang Y.J., Yan Z.J., Wang L.L., Meng Q.H., Yuan Y., Zhu G.S. (2018). Constructing synergistic groups in porous aromatic frameworks for the selective removal and recovery of lead (II) ions. J. Mater. Chem. A.

[76] Xu M.Y., Wang T., Gao P., Zhao L., Zhou L., Hua D.B. (2019). Highly fluorescent conjugated microporous polymers for concurrent adsorption and detection of uranium. J. Mater. Chem. A.

[77] Zhang C.R., Chen X.J., Niu C.P., Meng C., Yi S.M., Liu X., Qi J.X., Luo Q.X., Liang R.P., Qiu J.D. (2024). Reconstruction of biomimetic ionic channels within covalent organic frameworks for ultrafast and selective uranyl capture. Sci. China Chem..

[78] Li Z.N., Meng Q.H., Yang Y.J., Zou X.Q., Yuan Y., Zhu G.S. (2020). Constructing amidoxime-modified porous adsorbents with open architecture for cost-effective and efficient uranium extraction. Chem. Sci..

[79] Mollick S., Saurabh S., More Y.D., Fajal S., Shirolkar M.M., Mandal W., Ghosh S.K. (2022). Benchmark uranium extraction from seawater using an ionic macroporous metal–organic framework. Energy Environ. Sci..

