# Supplementary material for: Synergistic Adsorption and Fluorescence in Porous Aromatic Frameworks for Highly Sensitive Detection of Radioactive Uranium
Source: Molecules. 2025 Apr 25;30(9):1920. doi: 10.3390/molecules30091920 (PMC12073300; doi:10.3390/molecules30091920)
Supplement: Supplementary file 1 [file molecules-30-01920-s001.zip › molecules-3595298-supplementary.pdf]

Supporting Information

Synergistic adsorption and fluorescence in porous aromatic frameworks for highly sensitive detection of radioactive uranium

Suming Zhang [1], Siyu Wu [2], Cheng Zhang [2], Doudou Cao [2], Yingbo Song [2,*], Yue Zheng [2], Jiarui Cao [2], Lu Luo [2], Yajie Yang [3], Xiangjun Zheng [1,*] and Ye Yuan [2]

[1] Beijing Key Laboratory of Energy Conversion and Storage Materials, College of Chemistry, Beijing Normal University, Beijing 100875, China
[2] Key Laboratory of Polyoxometalate and Reticular Material Chemistry of Ministry of Education, Northeast Normal University, Changchun, 130024, China;
[3] Key Laboratory of Automobile Materials of Ministry of Education, School of Materials Science and Engineering, Jilin University, Changchun 130022, China

* Correspondence: Yingbo Song: songyingbo@nenu.edu.cn and Xiangjun Zheng: xjzheng@bnu.edu.cn

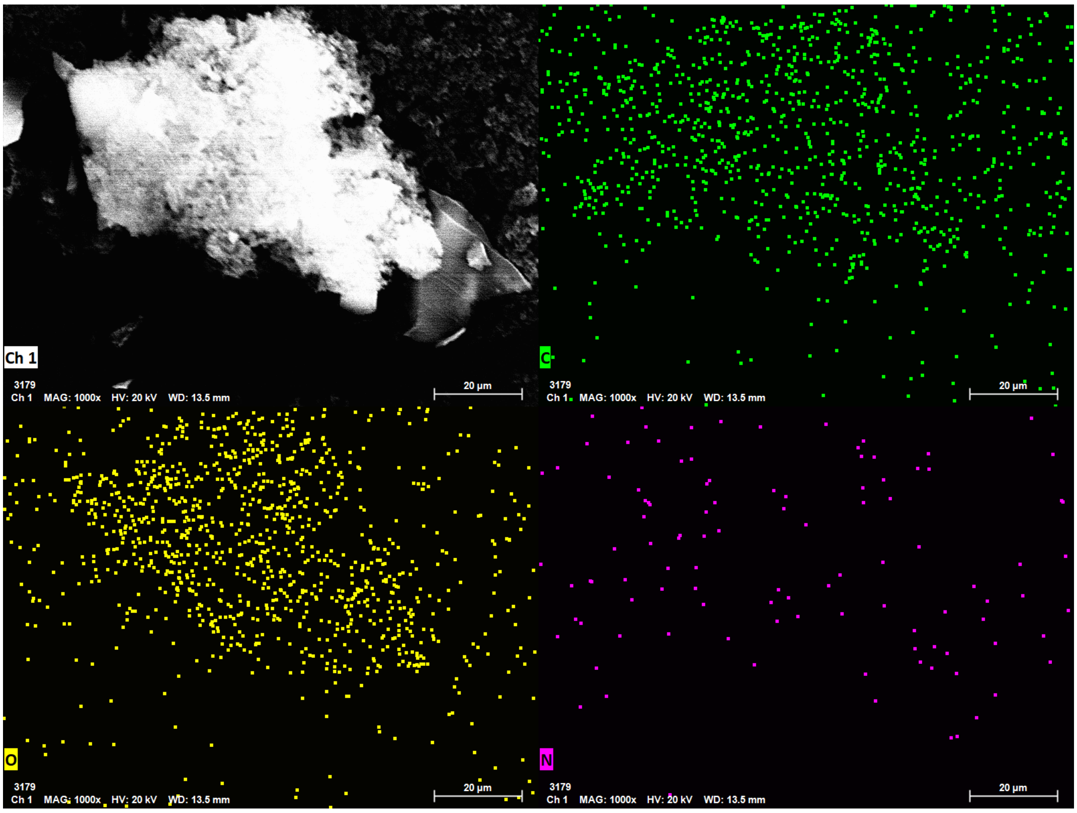

Figure S1: EDS images of MIPAF-15.

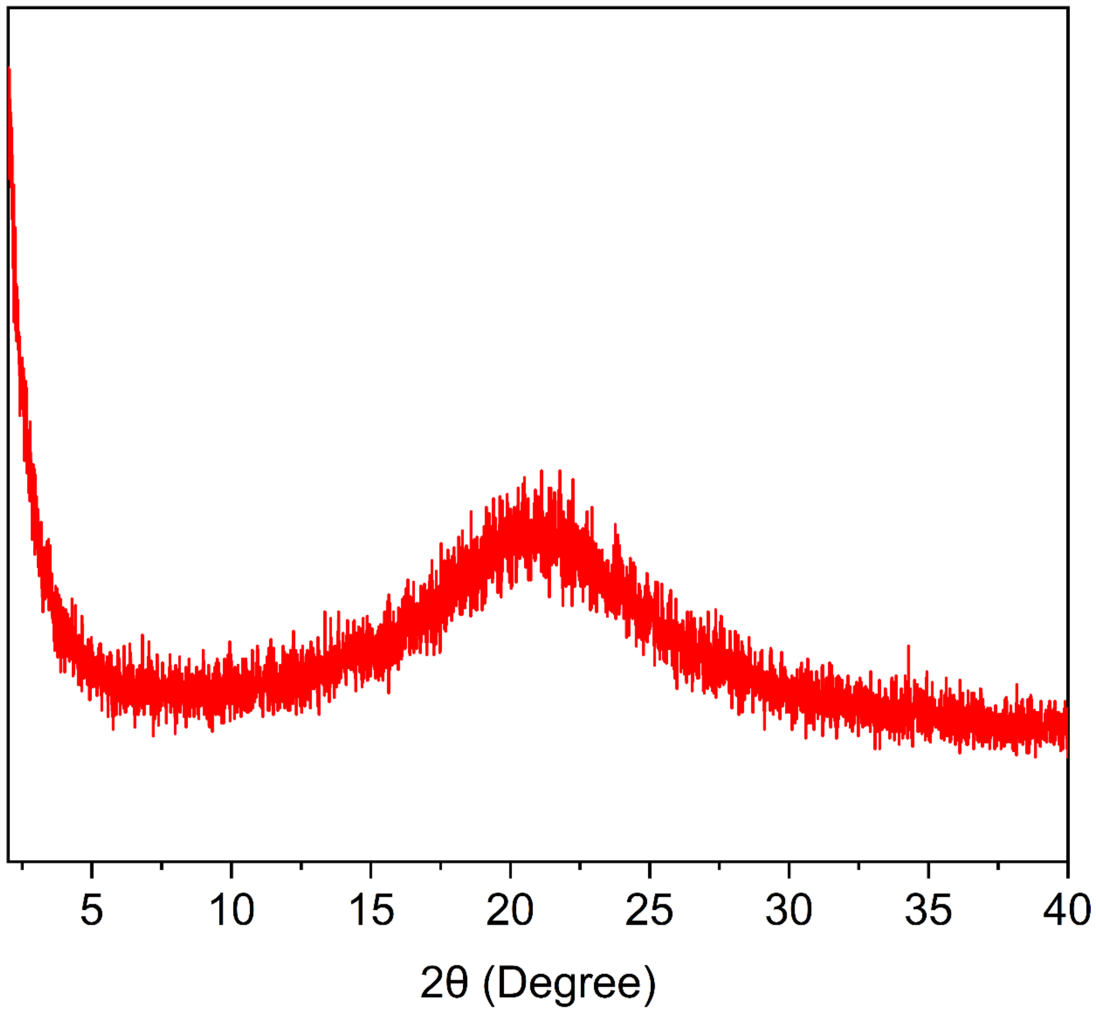

Figure S2: PXRD pattern of MIPAF-15.

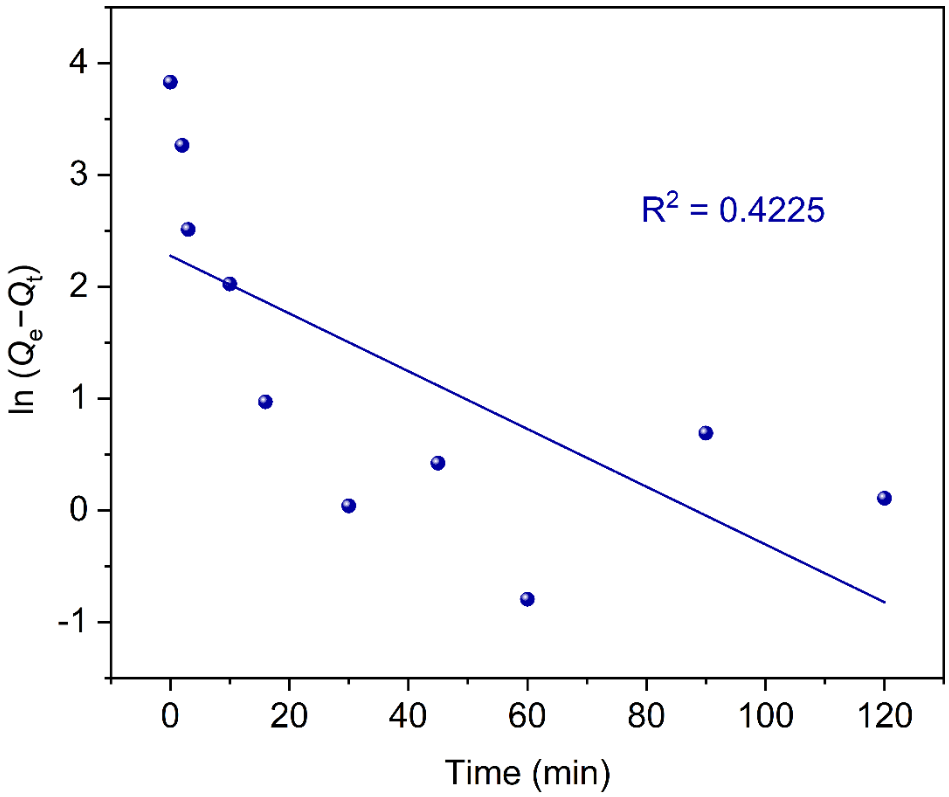

Figure S3: Kinetic fitting with *pseudo-first-order* model.

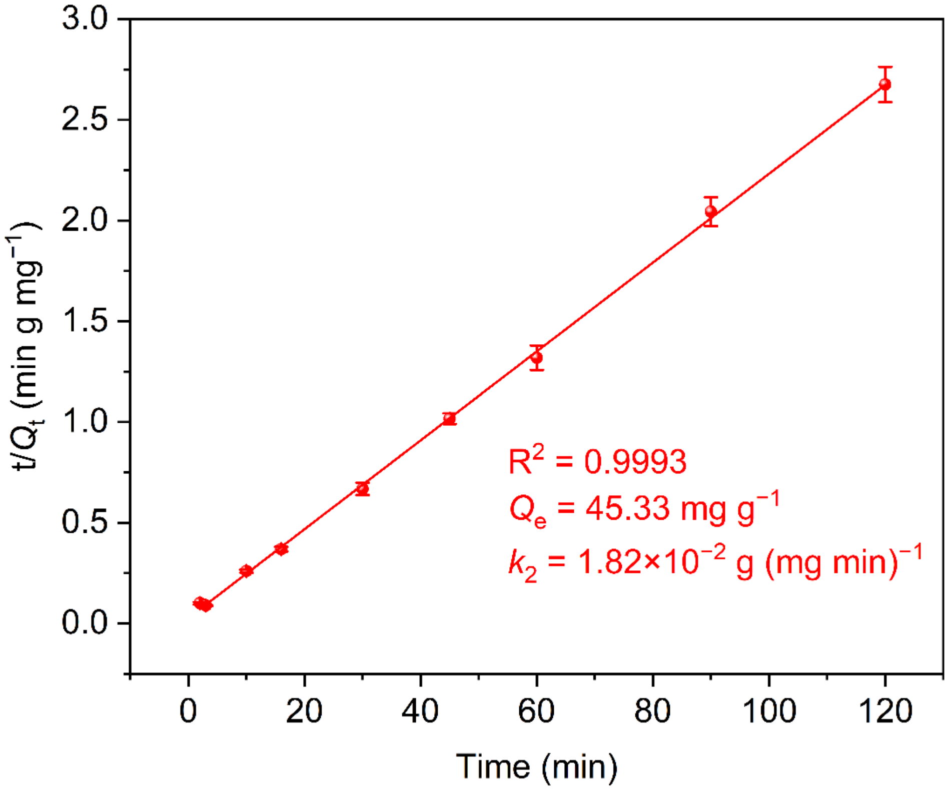

Figure S4: Kinetic fitting with *pseudo-second-order* model.

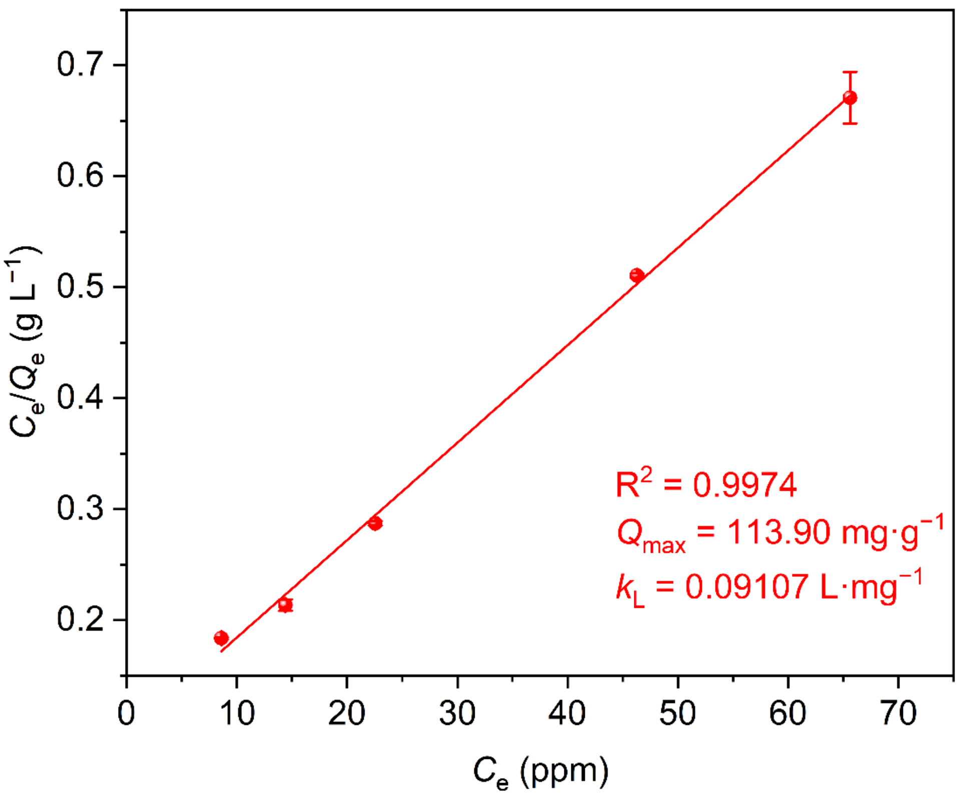

Figure S5: Fitting of adsorption isotherm with *Langmuir* model.

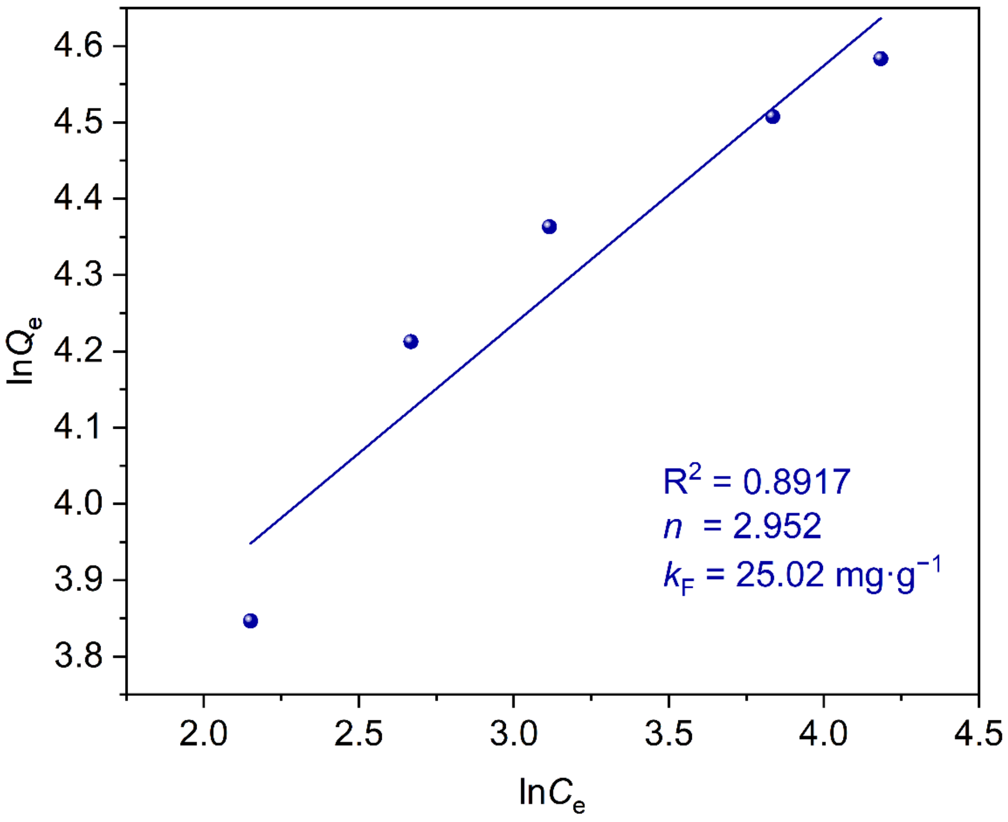

Figure S6: Fitting of adsorption isotherm with *Freundlich* model.

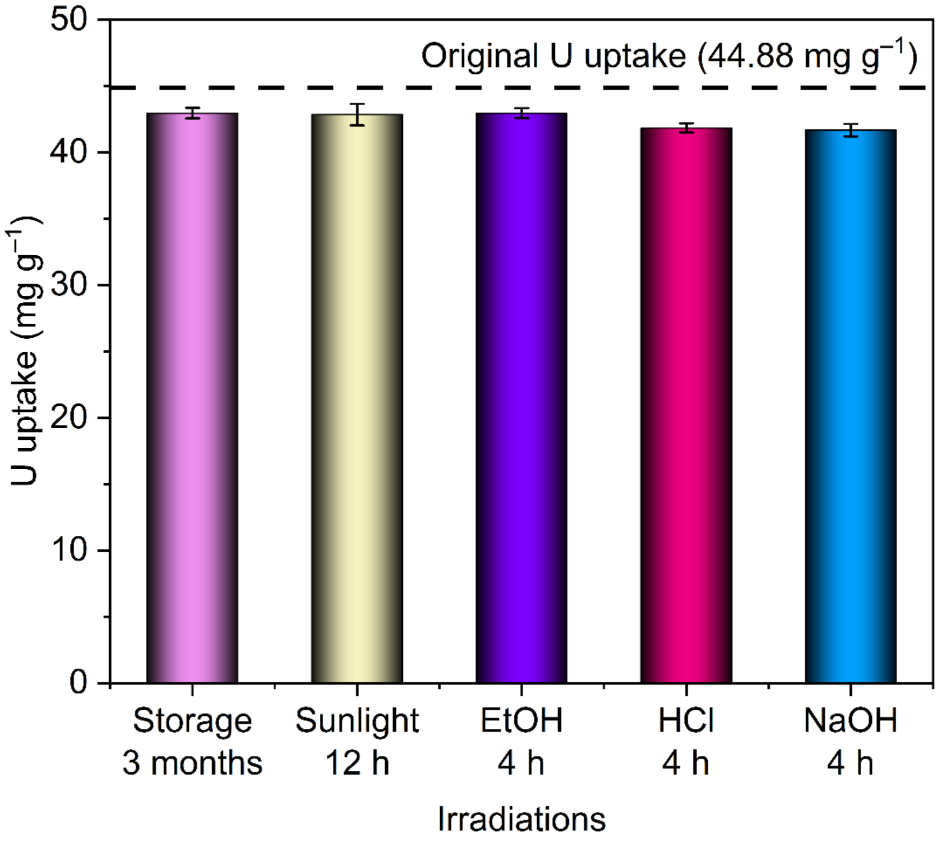

Figure S7: Stability tests of MIPAF-15 against different conditions. Pink: storage in air for 3 months; silver: irradiated by sunlight for totally 12 h; violet: soaked in ethanol for 4 h; magenta: soaked in HCl (1.00 M, aq) for 4 h; blue: soaked in NaOH (0.10 M) for 4 h.

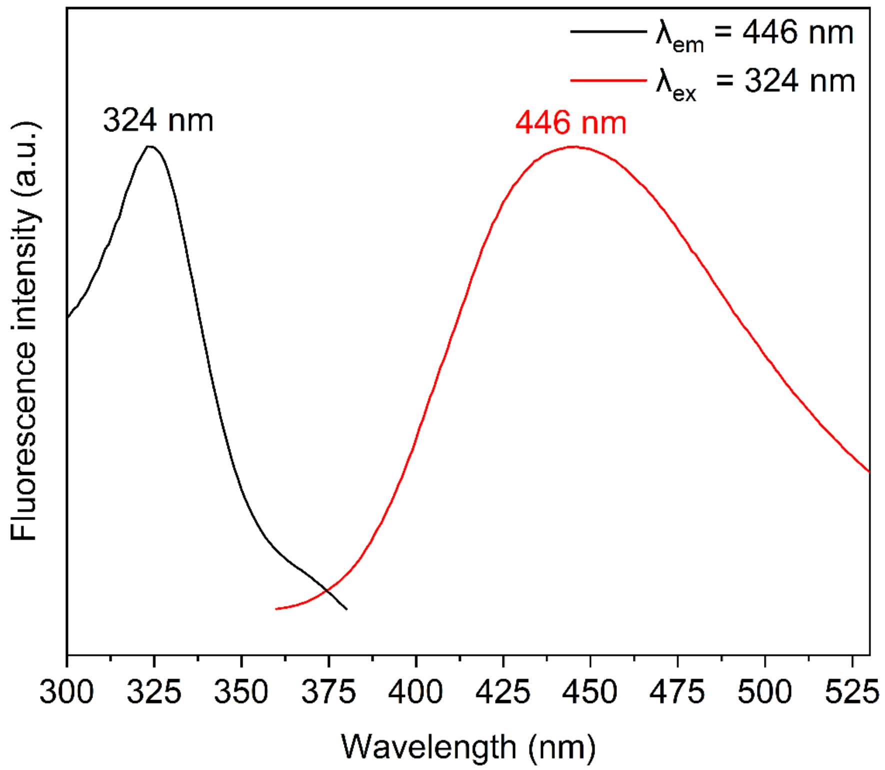

Figure S8: Fluorescence excitation and emission spectra of 4-bromo-1-*H*-indole-7-carboxylic acid.

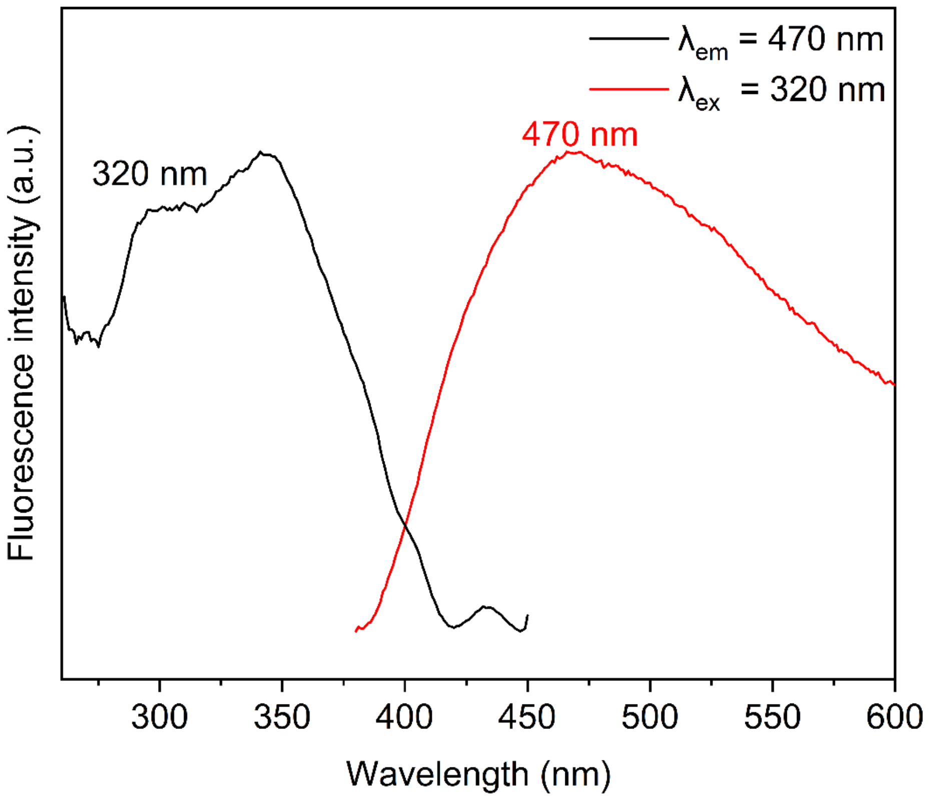

Figure S9: Fluorescence excitation and emission spectra of MIPAF-15.

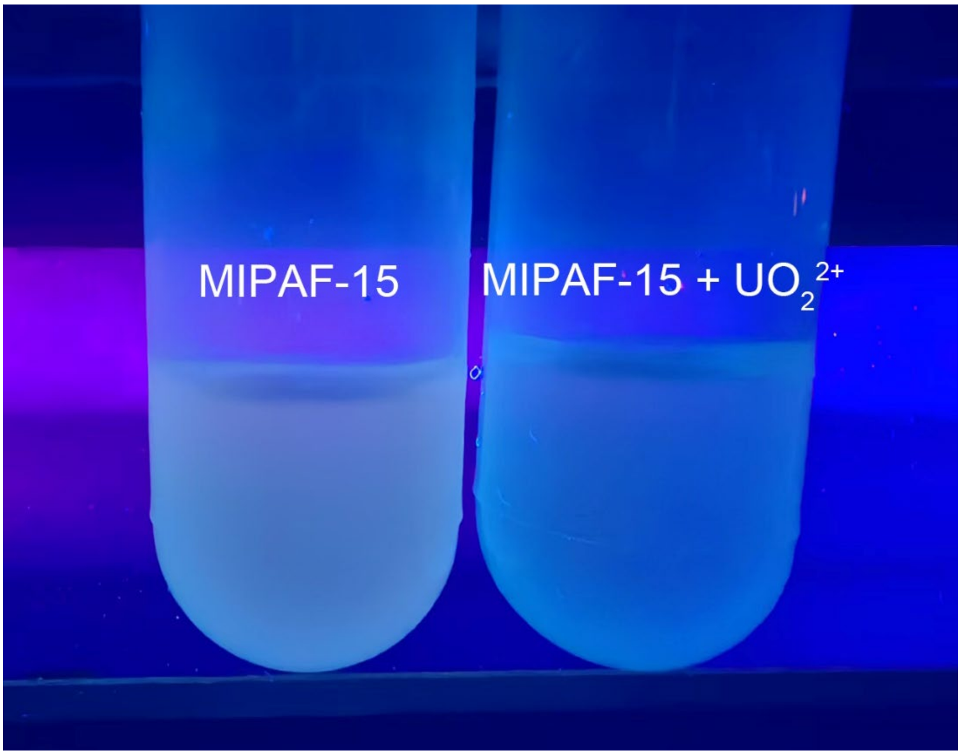

Figure S10: Photograph of MIPAF-15 with absence (left) and presence (right) of uranyl ions under 365 nm UV lamp

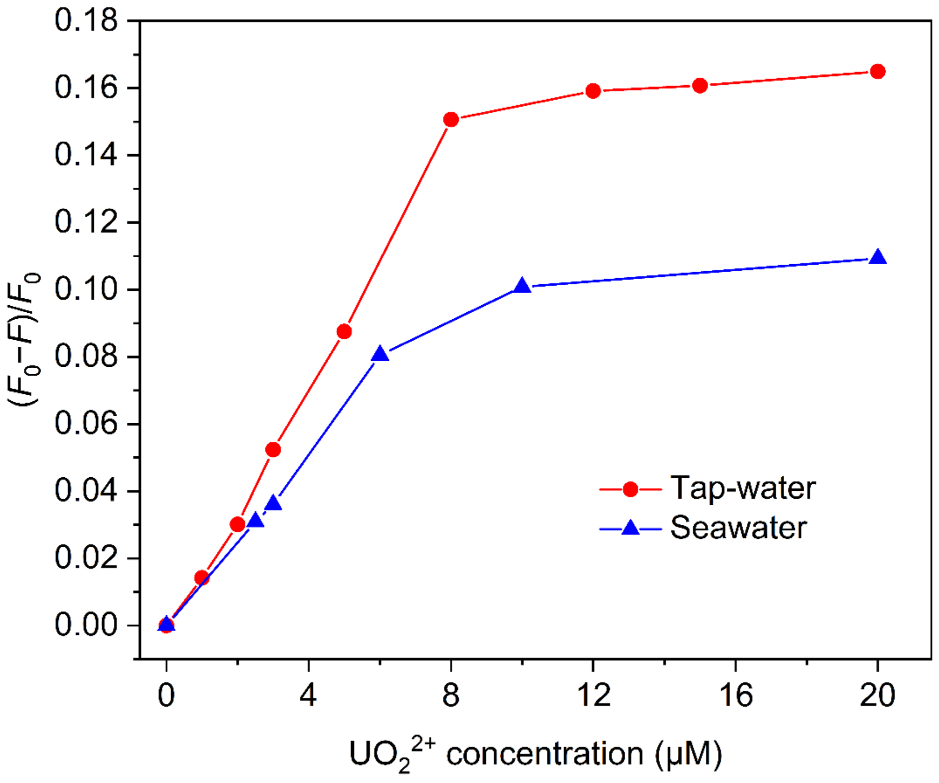

Figure S11: Correlation between fluorescence quenching ratio and uranyl ion concentration in selected natural water.

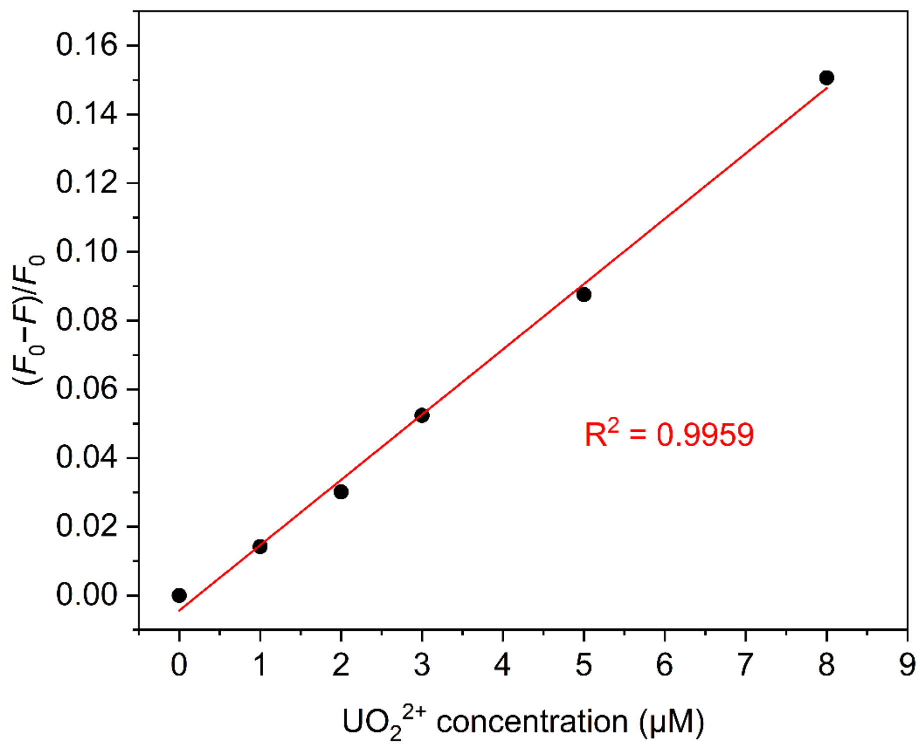

Figure S12: Linear fitting between fluorescence quenching ratio and uranyl ion concentration in tap-water.

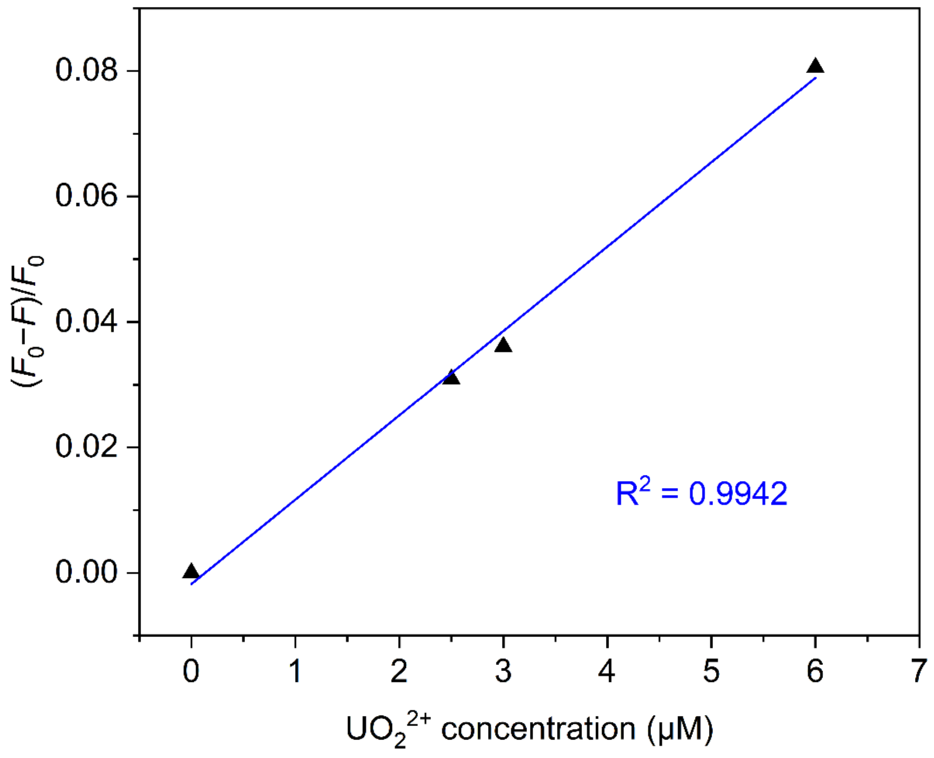

Figure S13: Linear fitting between fluorescence quenching ratio and uranyl ion concentration in seawater.

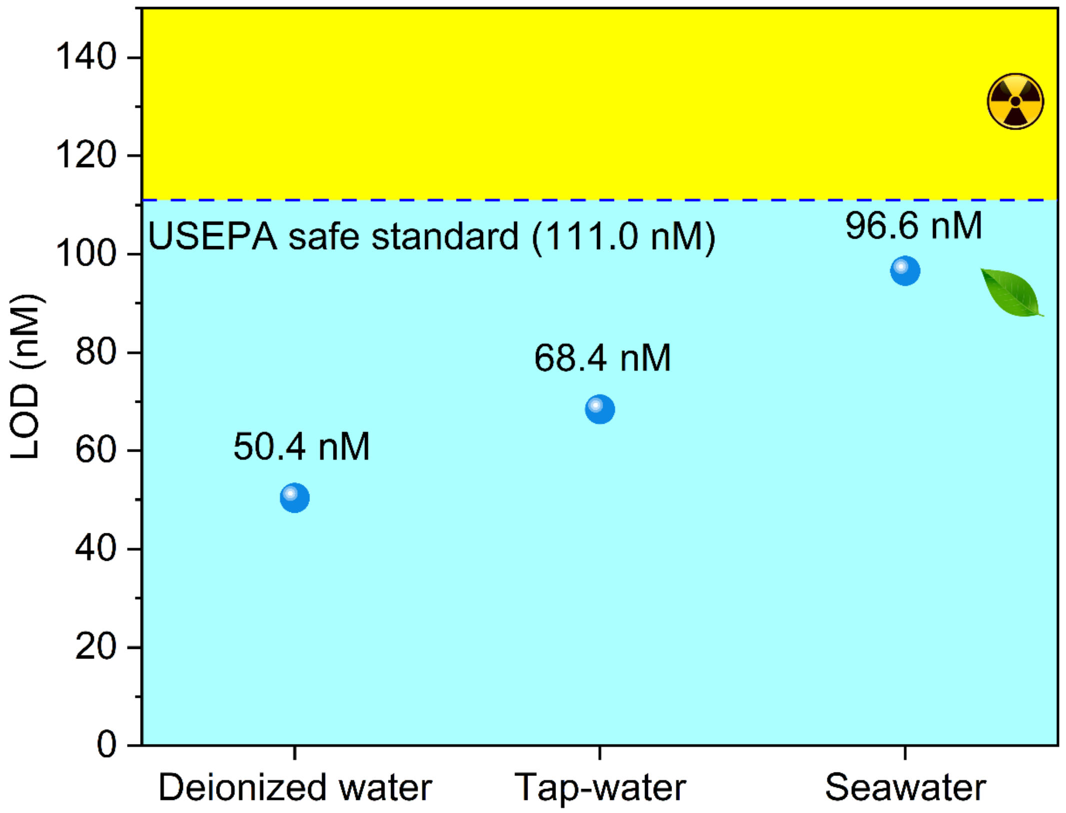

Figure S14: Detection limits of MIPAF-15 towards uranyl ions in different real water

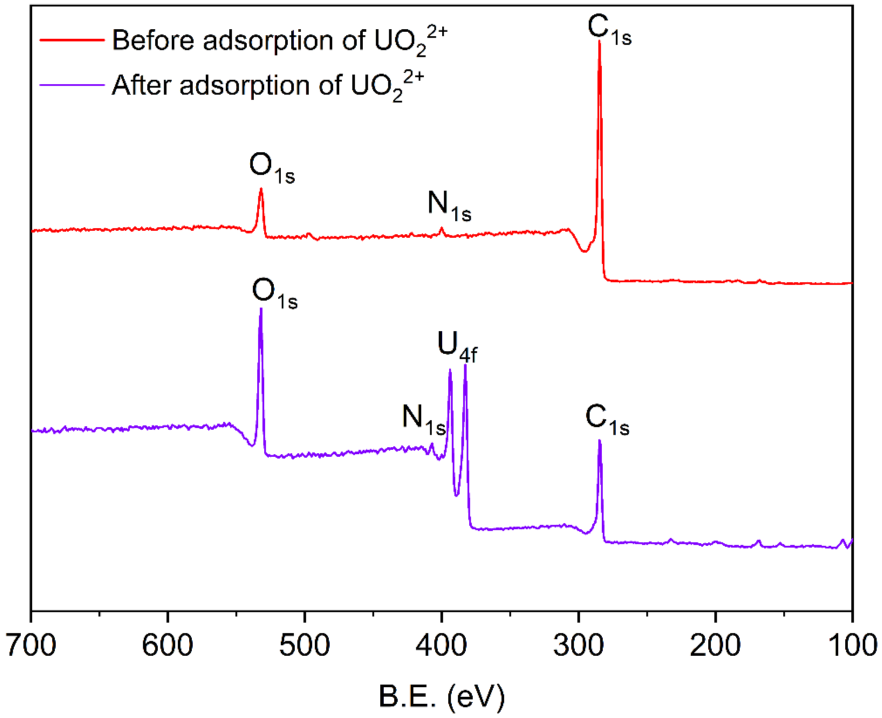

Figure S15: XPS spectra of MIPAF-15 before and after the adsorption of uranyl ions.

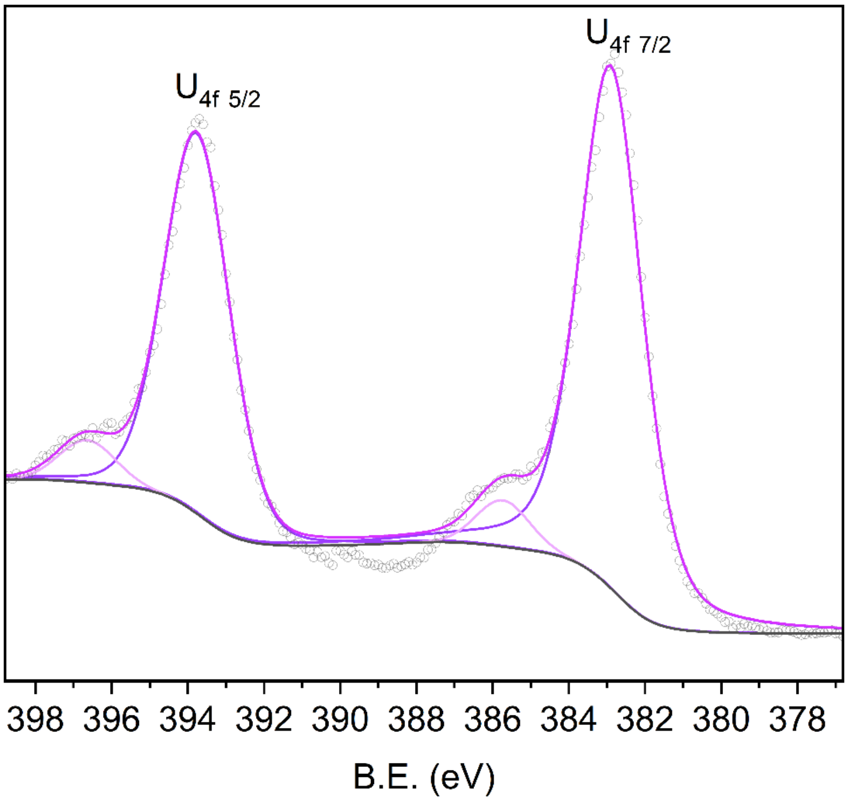

Figure S16: U_{4f} XPS spectra of uranyl-loaded MIPAF-15.

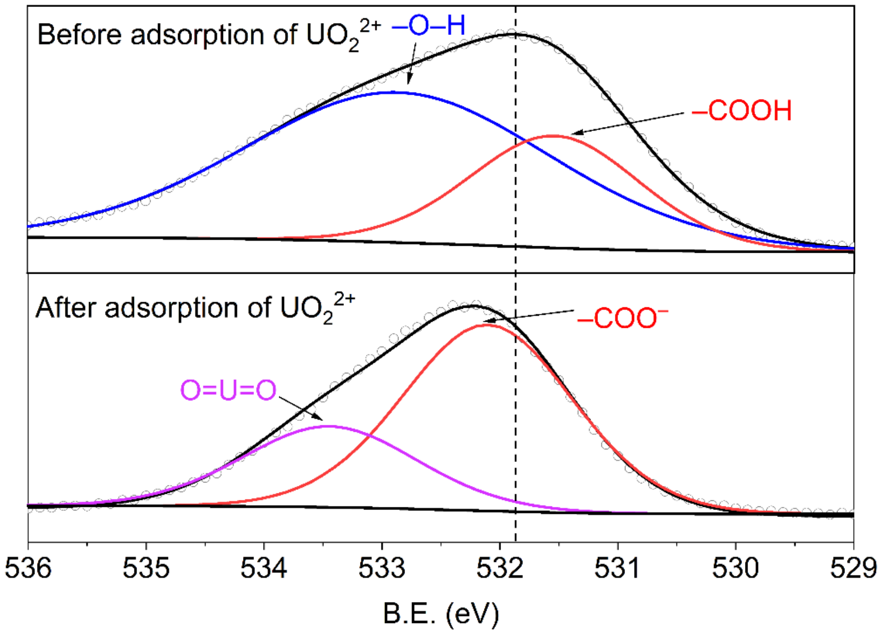

Figure S17: O_{1s} XPS spectra of MIPAF-15 before and after the adsorption of uranyl ions.

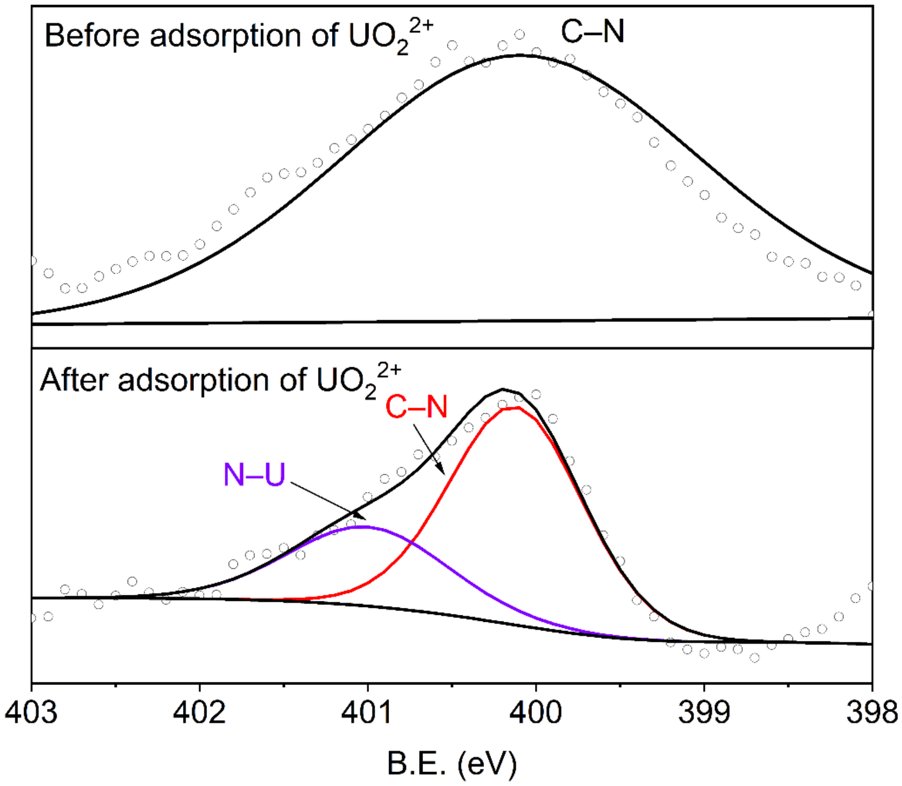

Figure S18: N_{1s} XPS spectra of MIPAF-15 before and after the adsorption of uranyl ions.
